# Resting State EEG in Exercise Intervention Studies: A Systematic Review of Effects and Methods

**DOI:** 10.3389/fnhum.2020.00155

**Published:** 2020-05-07

**Authors:** Mathias Holsey Gramkow, Steen Gregers Hasselbalch, Gunhild Waldemar, Kristian Steen Frederiksen

**Affiliations:** Department of Neurology, Danish Dementia Research Centre, Rigshospitalet, University of Copenhagen, Copenhagen, Denmark

**Keywords:** electroencephalography (EEG), exercise, intervention, power, LORETA (low resolution electromagnetic tomography), brain connectivity, asymmetry

## Abstract

**Background:** Exercise has been shown to alter brain plasticity and is explored as a therapeutic intervention in a wide variety of neurological diseases. Electroencephalography (EEG) offers an inexpensive method of studying brain electrocortical activity shortly after exercise and thus offers a way of exploring the influence of exercise on the brain. We conducted a systematic review to summarize the current body of evidence regarding methods of EEG analysis and the reported effects of exercise interventions on EEG.

**Methods:** PubMed, Web of Science and EMBASE were searched for studies investigating resting state EEG in exercise intervention studies carried out in participants >17 years of age and with no history of epilepsy. Further, studies solely investigating event-related potentials as an outcome measure were excluded. Relevant data were extracted, and a risk-of-bias assessment was carried out using the Cochrane risk-of-bias tool. A qualitative synthesis of results was carried out. A protocol for the systematic review was uploaded to https://www.crd.york.ac.uk/PROSPERO/ (ID: CRD42019134570) and the Preferred Reporting Items for Systematic Reviews (PRISMA) statement was followed.

**Results:** Out of 1,993 records screened, 54 studies were included in a final qualitative synthesis with a total of 1,445 participants. Our synthesis showed that studies were mainly carried out using frequency analysis as an analytical method. Generally, findings across studies were inconsistent and few were adjusted for multiple comparisons. Studies were mainly of low quality and usually carried out in small populations, lowering the significance of results reported.

**Conclusions:** Changes in the EEG as a result of an exercise intervention are elusive and difficult to replicate. Future studies should provide biologically sound hypotheses underlying assumptions, include larger populations and use standardized EEG methods to increase replicability. EEG remains an interesting methodology to examine the effects of exercise on the brain.

## Introduction

There is increasing evidence that exercise may have a profound effect on brain health (Barha et al., 2017). The connection between the brain and exercise is further corroborated by observed changes in brain circuits through altering of the synaptic plasticity after exercise, and possible induction of neurogenesis in the hippocampus by signaling molecules such as brain-derived neurotrophic factor and insulin-like growth factor-1 involved in these processes (Pedersen and Saltin, 2015; Cassilhas et al., 2016; Mellow et al., 2019). Exercise is currently being explored as a therapeutic option in a wide variety of brain diseases, and meta-analyses of findings have showed positive effects on anxiety and depression (Wegner et al., 2014). There is also evidence of a preventive effect of exercise on dementia (Pedersen and Saltin, 2015), while the therapeutic effect on manifest dementia such as Alzheimer's disease is more elusive and will need further research with better study designs (Frederiksen et al., 2018, 2019). One aspect of the influence of exercise on the brain is the idea of a runner's high, which is described as an anxiolytic, euphoric feeling, that is reported by long-distance runners (Hicks et al., 2019). The biochemical pathways underlying this phenomenon is currently being investigated, and reports point to an increase in plasma endocannabinoids that bind to cannabinoid receptors in the brain (Sparling et al., 2003; Fuss et al., 2015). Cannabinoid agonists have recently been shown to alter neural oscillatory activity (Skosnik et al., 2018), thus making it probable that changes in brain oscillations might be a reflection of this effect of exercise.

The sympathetic nervous system is activated during exercise (Christensen and Galbo, 1983). High intensity exercise leads to an increase in circulating cortisol immediately following exercise (Hill et al., 2008), which in resting individuals has been shown to affect brain oscillatory activity (Chapotot et al., 1998). Animal studies have recently shown that adrenergic modulation alters the dendritic excitability in cortical neurons in mice (Labarrera et al., 2018) and strengthens functional connectivity in the pre-frontal cortex in rhesus monkeys (Wang et al., 2007). This coupling between exercise, activation of the sympathetic nervous system and changes in the brain indicates that it might be feasible to measure an effect of exercise by recording the electrocortical activity in humans.

Some researchers link the beneficial effects of exercise to evolutionary features of humans, whereby exercise at a moderate intensity was necessary in hunter-gatherer communities and selection for this sort of activity has been pivotal in the evolution of the modern humans. This can teleologically explain the posited therapeutic effect of exercise in certain neurological disorders (Raichlen and Alexander, 2017).

EEG offers a non-invasive, cheap and easily applied method to study brain electrocortical activity (Rossini et al., 2019). As EEG can be applied shortly after an exercise intervention, it makes it possible to study temporary changes in electrocortical activity. However, it is difficult to capture, without massive influence of movement artifacts, the electrocortical activity *during* exercise, which represents a challenge for this field, although advances are being made to overcome this issue (Gwin et al., 2010). EEG analysis methods have also been developed to localize the anatomical brain substrate responsible for the signal that can be recorded from the scalp, thus enabling researchers to accurately localize the involved brain regions in e.g. exercise (Pascual-Marqui et al., 1994). Magnetoencephalography (MEG), although more costly to apply, has much of the same properties as EEG, and may be superior in source localization (Cohen and Cuffin, 1983). MEG has a preference for capturing cortical activity in the sulcis of the brain by the recording of tangential electromagnetic fields generated in the sulcal walls (Baillet, 2017). Both EEG and MEG thus offer unique methods for exploring the possible effect of exercise on the brain. Another mentionable method of investigation of brain-exercise dynamics is functional near-infrared spectroscopy (fNIRS) (Herold et al., 2018). Although beyond the scope of this review, this method offers good temporal resolution of brain oxygenation and hemodynamics related to exercise and could be joined with EEG/MEG for assessing hemodynamic associations with phenomena recorded using EEG/MEG (Herold et al., 2018).

To our knowledge, no systematic review exists on the effects of exercise interventions on EEG or MEG. Previous reviews have focused on the affective part of exercise (Lattari et al., 2014) and another review mainly included studies that could be assessed by meta-analytic methods (Crabbe and Dishman, 2004). We thus conducted a comprehensive systematic review to summarize (1) the current body of evidence regarding the effects observed in exercise intervention studies and (2) analytical methods used to quantify these.

### Rationale

Since exercise may alter brain plasticity and possibly brain circuits, which might be reflected in EEG or MEG recordings, we wanted to search the literature for studies reporting on EEG or MEG in exercise interventions.

### Objectives

To summarize studies reporting on the effects on the EEG/MEG and methods used to analyze resting state EEG/MEG in exercise intervention studies in healthy and diseased individuals.

### Research Question

What are the effects of exercise EEG/MEG derived measures of brain activation and by what methods is the EEG/MEG analyzed in exercise intervention studies?

## Methods

### Study Design

We performed a systematic review in accordance with the guidelines provided by the Preferred Reporting Items in Systematic Reviews (PRISMA) statement (Moher et al., 2009).

### Participants, Interventions, Comparators

We included single group, cross-over or parallel group studies with both randomized and non-randomized allocation involving participants who were above the age of 17 years, with no history of epilepsy or sleep disorders. Only full research articles were included. Exercise interventions could be either acute (single bout) or chronic exercise interventions (≥2-weeks of duration). The outcome assessed was changes in the EEG signal or changes in the MEG, which could be analyzed in any way. We excluded studies involving event-related potentials and studies involving sleep EEG.

### Systematic Review Protocol

A systematic review protocol was registered 30th of August 2019 in the PROSPERO database (PROSPERO ID: CRD42019134570) (https://www.crd.york.ac.uk/prospero/).

### Search Strategy

A search for (“EEG” OR “MEG”) AND “exercise” was carried out. Detailed search strings for the three databases searched are uploaded as Supplementary Material.

### Data Sources, Study Selection, and Data Extraction

We searched PubMed (MEDLINE), EMBASE, and Web of Science for records using the previously mentioned search strings. Final searches were conducted 25th of September 2019. Two of the authors, KF and MG, independently screened titles and abstracts and full-text articles. Any disagreements were resolved by discussion and no third party was involved in the selection of studies. Relevant data was extracted by the same authors (MG and KF) using an Excel data extraction sheet that was piloted in three studies before being applied to the rest of the studies. The following items were extracted from the studies:

Study design, comparator (if applicable), age and sex of participants, number of participants, intervention characteristics (type of intervention, length of intervention, intensity of aerobic exercise), EEG/MEG methods, EEG/MEG software, EEG/MEG metrics (e.g., connectivity, modulation of frequency), EEG/MEG paradigm, statistical analysis methods, diagnosis of participants, reported effect of the intervention on EEG/MEG.

A risk-of-bias assessment using version 2 of the revised Cochrane risk-of-bias tool for randomized trials was carried out on included studies (Sterne et al., 2019).

### Data Analysis

A qualitative synthesis of results was done as according to protocol. Due to expected large heterogeneity in the studied outcomes, interventions and populations, we did not plan to carry out a meta-analysis nor was this done as a *post-hoc* analysis due to the aforementioned reasons. Throughout the manuscript, reported results from included studies are only defined as statistically significant if the included study reported a *P* < 0.05 for the finding. Additionally, we extracted data on whether studies adjusted for multiple comparisons (Benjamini-Hochberg, Bonferroni-Holm, etc.) and denoted studies that specifically reported this.

## Results

### Study Selection and Characteristics

**Figure 1:**
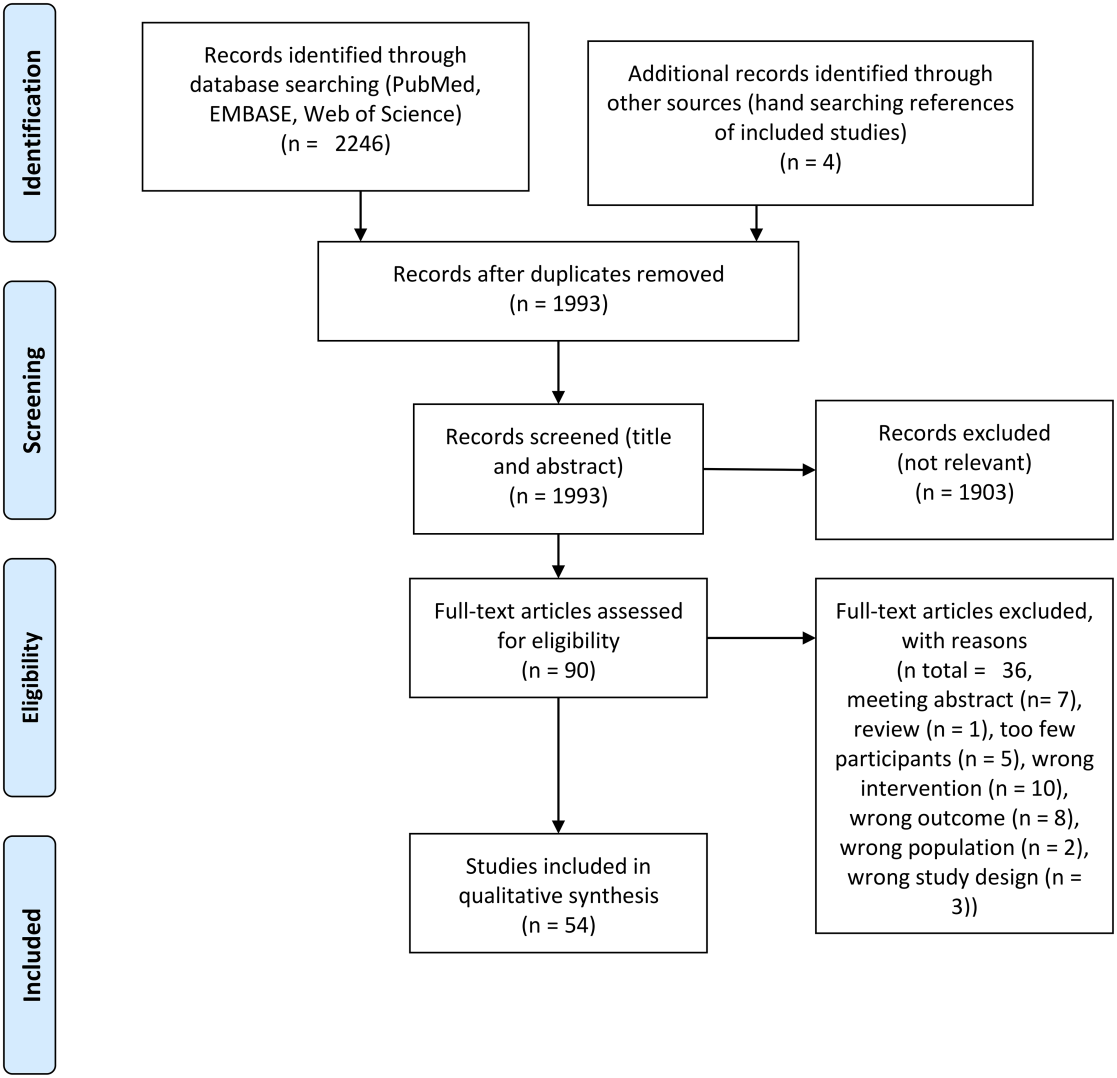
PRISMA flow chart of included studies.

The study selection process is outlined in Figure 1. A total of 2,250 records were identified through bibliographic searches and hand searches of included studies from which 54 studies with a complete total of 1,445 participants were included. Characteristics of the included studies are shown in Table 1. Of the 54 studies included, 43 studies included subjects who were younger than 50 years of age and 43 studies included participants who were healthy. In total, 40 studies investigated the acute effect of exercise. The study designs of the included studies were as follows: Single group (*n* = 20), parallel group (*n* = 5), cross-over (*n* = 12; of these, *n* = 4 were sequence randomized), non-randomized, controlled (*n* = 6), randomized, non-controlled (*n* = 5) and randomized, controlled (*n* = 7). In total, 22 studies were done on <20 participants. Four studies performed adequate statistical multiple comparison adjustment (Spring et al., 2017, 2018; Hübner et al., 2018; Devilbiss et al., 2019; Villafaina et al., 2019). It should be noted that these four studies were published recently. No studies reporting on MEG were found in our searches. Table 2 outlines elaborated EEG methods in the included studies.

**Table 1:** Characteristics of the included studies.

**Healthy persons** **<** **50 years, acute intervention**							
**References**	***N* (female percentage if stated)**	**Mean age (sd) for whole cohort if not stated otherwise**	**Study design**	**Intervention**	**Participants**	**EEG paradigm**	**Main results**
Bailey et al. (2008)	20 (0)	24 (1.5)	Single group	Graded, until volitional exhaustion, recumbent cycle ergometer	Healthy	Initially 21, but focused on 8 leads (F3, F4, F7, F8, C3, C4, P3, P4) in the final analysis, frequency analysis, alpha/beta ratio	Across all leads increases immediately after exercise in theta, alpha-1, alpha-2, beta-1 and beta-2, that returned to baseline within 10 min after exercise. Significant increases in alpha/beta ratio in frontal leads that remained significant only for F7 and F8 10 min post-exercise
Bixby et al. (2001)	27 (51.8)	23.3 (3.5)	Single group	Two times 30 min exercise, High intensity: Ventilatory aerobic breakpoint, low intensity: 75% of ventilatory breakpoint, cycle ergometer	Healthy	F8-F7, F4-F3, P4-P3, frequency analysis, alpha asymmetry score	Frequency analysis: Parietal alpha power increase. Asymmetry analysis: No significant changes
Boutcher and Landers (1988)	30 (0)	Runners: 29.9 (9) Non-runners: 26.7 (4.6)	Cross-over	20 min, 80–95% of max heart rate, treadmill	Healthy runners	T3, T4, frequency analysis	Significant bilateral alpha power increase within the first 14 min after running for both groups
Brümmer et al. (2011b)	26 (42.3)	26 (6)	Single group	Graded, until subjective exhaustion, cycle ergometer	Healthy regular cyclists, cycling once per week for recreation or transport	Fp1, Fp2, F7, F3, Fz, F4, F8, FC5, FC1, FC2, FC6, T7, C3, Cz, C4, T8, TP9, CP5, CP1, CP2, CP6, TP10, P7, P3, Pz, P4, P8, PO9, O1, Oz, O2, PO10, LORETA	Sensory motor cortex current density decreased
Ciria et al. (2018)	20 (0)	23.8	Cross-over	30 min flanked by 10 min 20% VO_2_ max warm-up and cool-down, two conditions: low (20% VO_2_ max) and moderate (80% VO_2_ max), cycle ergometer	Healthy	30 electrodes, frequency analysis, current source density	Power spectrum and current source density analysis: There were no significant differences between low and moderate intensity resting states. T-test statistics were corrected for multiple comparisons, but results were not corrected for number of groups and frequency bands
Devilbiss et al. (2019)	16 (50)	Men: 19.9, women: 19.6	Single group	1 mile, individual all-out (5–10 min), grass track running	Healthy soccer athletes	1 electrode near position Fp1, frequency analysis	Lower relative theta power. Other frequencies insignificant changes. Benjamini-Hochberg false discovery rate adjusted
Fumoto et al. (2010)	10 (10)	32 (2.2)	Single group	15 min, Borg scale = 12–13, cycle ergometer	Healthy, light exercisers	Cz, Fz, frequency analysis	Significant decrease in theta for central and frontal leads, significant increase in alpha-2 for central and frontal leads. No significant changes found in alpha-1 or in beta
Teixeira Guimaraes et al. (2014)	10 (0)	20–27 (range)	Randomized, cross-over	Submaximal: 30–62 min at +-9% VO_2_ max, Maximal: Graded until VO_2_ plateau ≤ 150 mL/min or 2 kg mL/min, heart rate ≥ 90% predicted by age (220-age), Borg scale ≥ 18 or ≥ 1.15 respiratory exchange ratio, and voluntary failure to maintain the cadence. Supramaximal: 30 s sprint against a workload of 0.075 kp body mass(1/kg), cycle ergometer for all intensities	Healthy, regular exercisers	Fz, Cz, Pz, Oz, Fp1, Fp2, F3, F4, F7, F8, C3, C4, T3, T4, T5, T6, P3, P4, O1, O2, sLORETA	Significant increases in maximal effort for alpha-2 and beta-2 in Brodmann area 27 (parahippocampal gyrus-limbic lobe), and beta-2 in Brodmann area 19 (parahippocampal gyrus-limbic lobe). *T*-test statistics were corrected for multiple comparisons, but results were not corrected for number of groups and frequency bands
Gutmann et al. (2018b)	Experiment 1: 97 (Group 1: 21, 2: 26, 3: 40, 4: 33), Experiment 2 95 (Group 1: 28, 2: 28, 3: 38, 4: 33)	Experiment 1 (Group 1: 23.4 (3.6), 2: 23.8 (3.5), 3: 24.3 (3.8), 4: 24.3 (3.8), Experiment 2 (Group 1: 1: 23.84 (3.79), 2: 23.68 (3.33), 3: 23.9 (2.28), 4: 24.2 (3.89)	Parallel group	Two-part experiment: participants all did a graded exercise test until volitional exhaustion, then four groups were made with different resting times: no rest, 30 min, 60 min and 90 min. Following rest, participants were then divided into four groups of 30 min low (45–50% of maximum heart rate), moderate (65–70%), high intensity (85–90%) and control, cycle ergometer	Healthy	Fp1, Fp2, F3, Fz, F4, T7, C3, Cz, C4, T8, P3, Pz, P4, O1, O2, peak alpha frequency	All groups' alpha peak shifted to a higher frequency immediately after graded exercise but was unchanged 30 min after this intervention. Alpha peak shifted to higher frequency only after high intensity exercise and returned to baseline values 20 min thereafter
Hicks et al. (2018)	12 (58)	22.3 (3.1)	Randomized, cross-over	30 min, at 60–70% maximum heart rate and movement intervention consisting of pedaling againts no resistance, recumbent cycle-ergometer	Healthy	32-channel cap, only used F3 and F4, frequency analysis, frontal alpha asymmetry	Alpha frontal asymmetry significantly larger at 22 and 30 min after exercise compared to pre but not immediately after exercise. Frontal alpha power increased significantly and stayed increased for whole follow up period after exercise and for 6 min after movement intervention
Hilty et al. (2011)	17 (0)	25.9 (3.5)	Single group	Graded, until volitional exhaustion, home trainer	Healthy, regular endurance exercisers	128-channel HydroCel Geodesics Sensor Net, sLORETA, mean lagged synchronization in alpha band	Mean alpha power and lagged synchronization were unchanged. Eyes closed: Increase in alpha and beta band activity in Brodmann area 11. *T*-test statistics were corrected for multiple comparisons, but results were not corrected for number of groups and frequency bands
Hottenrott et al. (2013)	16 (0)	25.9 (3.8)	Single group	60 min, 90% lactate threshold, cycle ergometer	Healthy, endurance cyclists	Fp1, Fp2, F7, F3, Fz, F4, F8, FC5, FC1, FC2, FC8, T7, C3, Cz, C4, T8, TP9, CP5, CP1, CP2, Cp6, TP10, P7, P3, Pz, P4, P8, PO9, O1, Oz, O2, PO10, frequency analysis	Theta, alpha-1, alpha-2, beta-1, and beta-2 decreased
Kubitz and Mott (1996)	34 (41)	23.4 (3.7)	Non-randomized, controlled	15 min, with each 5 min increment adjusted to an initial 50 W load heart rate, cycle ergometer	Healthy	F3, F4, T3, T4, frequency analysis	No significant changes
Kubitz and Pothakos (1997)	28 (46)	21.7 (2.03)	Randomized, controlled	15 min, at a heart rate of 145–160 bpm, cycle ergometer	Healthy	F3, F4, T3, T4, frequency analysis	No significant differences between or within groups between exercise and recovery period. A significant time effect was found, but included measurements during a vigilance task, so does not clarify whether exercise was the main driver behind this effect
Lattari et al. (2016)	20	26.5 (3.8)	Cross-over	Two interventions and one control visit: Prescribed exercise (PE): 20 min, 50% VO_2_ max; Self-selected exercise (SS): 20 min, individually selected tempo, for PE and SS: cycle ergometer	Healthy, physically active (exercised aerobically 3 times weekly)	Fp1, Fp2, Fz, F3, F4, F7, F8, Cz, C3, C4, T3, T4, T5, T6, Pz, P3, P4, Oz, O1 and O2, but only analyzed F3-F4, frontal alpha asymmetry	No significant changes
Mechau et al. (1998)	19 (10.5)	42.4 (8.3)	Single group	Graded, until volitional exhaustion, track running	Healthy, leisure-time athletes	17 electrodes, frequency analysis	Significant increases for delta, theta, alpha-1, alpha-2, beta-1, beta-2 after each 6 min stage of exercise, except for alpha-2 (stage 1) and beta-2 (stage 4 and 5). All except delta returned to baseline values within 15 min after last stage
Mierau et al. (2009)	30 (0)	26 (4)	Three-arm, randomized	Three interventions, running, tracking task (non-aerobic) and both. Exercise intervention was graded, until volitional exhaustion, treadmill	Healthy runners	Fp1, Fp2, Fz, F3, F4, C3, C4, Cz, P3, P4, Pz, F7, F8, T7, T8, P7, P8, O1, O2, frequency analysis	No significant changes
Moraes et al. (2007)	10 (60)	25.6 (4.1)	Single group	Graded, until volitional exhaustion, Borg scale ≥18, heart rate ≥90% maximal heart rate, or incapacity to continue the test, cycle ergometer	Healthy, cycle ergometer exercisers	20 electrodes, frequency analysis	Increased beta power (Fp1, F3, F4 and C4). No effect on alpha power
Mott et al. (1995)	33 (42)	23.4 (3.7)	Non-randomized, controlled	15 min, 50% VO_2_ max, cycle ergometer	Healthy	4 electrodes, placed frontally and temporally, coherence analysis	Authors used different methods (1–4) for data segmentation. Intervention group: For method 1 and method 2 significant increases in alpha coherence. No significant changes were observed for beta. Controls: significant reduction in beta coherence in the right hemisphere
Ohmatsu et al. (2014)	16 (50)	Intervention 23.5 (1.9), control: 23.1 (1.9)	Non-randomized, controlled	30 min, 50% of VO_2_ max, cycle ergometer	Healthy	Fp1, Fp2, AF3, AF4, F7, F3, Fz, F4, F8, FC5, FC1, FC2, FC6, T7, C3, Cz, C4, T6, CP5, CP1, CP2, CP6, P7, P3, Pz, P4, P8, PO3, PO4, O1, Oz, O2, frontal asymmetry and sLORETA	sLORETA: Alpha-2 anterior cingulate cortex decrease. Asymmetry: Frontal alpha-1 asymmetry increased
Petruzzello and Landers (1994)	20 (0)	22.7 (2.4)	Single group	30 min, 75% of maximal aerobic capacity, treadmill	Healthy, regular exercisers	F3, F4, T3, T4, frequency analysis	No significant changes
Petruzzello and Tate (1997)	20 (25)	22.6 (3.3)	Randomized, cross-over	30 min, 55 and 70% VO_2_ max, cycle ergometer	Healthy, regular exercisers	F3, F4, P3, P4, frequency analysis, frontal alpha asymmetry	No significant changes
Pineda and Adkisson (1961)	16	22–36 (range)	Single group	Graded, until volitional exhaustion, treadmill	Healthy	6 electrodes, alpha index	Greater alpha activity in frontal compared to central, as well as greater alpha activity in central compared to occipital region. No significance testing
Schneider et al. (2009a)	24 (37.5)	30.1 (7.6)	Single group	Graded, 50–55, 80–85% VO_2_ Max and preferred, outdoors running	Healthy runners	Fp1, Fp2, F3, F4, F7, F8, Fz, C3, C4, Cz, P3, P4, P7, P8, Pz, T7, T8, O1, O2, frequency analysis and frontal mean spectral asymmetry	Frequency analysis: For low intensity: increased alpha-1 immediately post compared to pre, for preferred and high intensity: decrease in beta-2 immediately and 15 min post intervention. Alpha-1 activity was driven by occipital and frontal leads, whereas beta-2 was driven by frontal, parietal, central and occipital leads. Asymmetry score: No significant changes
Schneider et al. (2009b)	12 (33.3)	26.3 (3.8)	Cross-over	Graded, until volitional exhaustion, treadmill, arm-crank and cycle ergometer	Healthy runners, 2 h per week minimum	Fp1, Fp2, F3, F4, F7, F8, Fz, C3, C4, Cz, P3, P4, P7, P8, Pz, T7, T8, O1, and O2, sLORETA	Arm crank: Alpha activity increased in one voxel in frontal lobe (Brodmann area 45), beta increase in parietal lobe (Brodmann area 7 and 40) immediately after intervention, alpha and beta activity in left and right temporal lobes were increased up to 15 min after, beta activity was increased in limbic area (Brodmann area 30/31) 30 min after.
							Treadmill: Frontal (Brodmann area 6, 8 and 9) and limbic (Brodmann area 24 and 32) alpha increase, and parietal (Brodmann area 7) beta increase, which were not significant 15 min post intervention. Bike: Significantly increased alpha in parietal (Brodmann area 7) and limbic (Brodmann area 23 and 31) areas immediately after and in frontal (Brodmann are 6 and 9) and limbic (Brodmann area 24 and 32) areas 15 min post intervention, with no significant beta changes. *T*-test statistics were corrected for multiple comparisons, but results were not corrected for number of groups and frequency bands
Schneider et al. (2010a)	22 (35.4)	30.6 (7.7)	Single group	Graded, until volitional exhaustion, treadmill	Healthy runners, minimum of 2 h per week	Fp1, Fp2, F3, F4, F7, F8, Fz, C3, C4, Cz, P3, P4, P7, P8, Pz, T7, T8, O1, and O2, sLORETA	Left middle frontal gyrus alpha-1 increase, widespread increase in delta, which lasted at least 15 min and left and right temporal theta activation, other frequencies were not significant immediately post exercise vs. pre. Decrease in alpha-2 activity left inferior temporal gyrus (one voxel), beta-1 decrease left inferior, middle and superior temporal gyri, reduction in gamma activity in left part of cuneus 15 min post exercise vs. pre. *T*-test statistics were corrected for multiple comparisons, but results were not corrected for number of groups and frequency bands
Schneider et al. (2010b)	18 (33.3)	28.8 (6.0)	Randomized, cross-over	Duration not stated, low (50–55% VO_2_ max), high (80–85% VO_2_ max) and preferred intensity, track running	Healthy runners	Fp1, Fp2, F3, F4, F7, F8, Fz, C3, C4, Cz, P3, P4, P7, P8, Pz, T7, T8, O1, and O2, sLORETA	Significant delta activity increase in frontal and limbic lobe areas after high, but not low or preferred exercise. *T*-test statistics were corrected for multiple comparisons, but results were not corrected for number of groups and frequency bands
Spring et al. (2017)	20 (0)	30.8 (6.9)	Cross-over	Intervention 1: 30 min, 60% of maximal aerobic power, followed by intervention 2: 10 km time trial, graded, all-out, 1: Cycle ergometer, 2: home trainer	Healthy road cyclist/triathletes	Fp1, Fpz, Fp2, C1, Cz, C2, PO3, POz, PO4, frequency analysis, microstate analysis	Frequency analysis: delta decreased after intervention 1. Theta, alpha and beta power increased after intervention 2, compared to pre. Microstate analysis: Global variance explained, mean duration of, and time coverage for microstate class C were all significantly increased after intervention 1 and 2 compared to the pre-intervention resting state. No significant transition patterns were found from which the microstate changed from to state C. Results were corrected for multiple comparisons by Bonferroni
Spring et al. (2018)	42 (57.9)	24 (4)	Single group	Graded, until volitional exhaustion followed by 25 min of Borg scale 15, cycle ergometer	Healthy, physically active	64 channels (EASYCAP), microstate analysis	Microstate B and C mean duration was increased and stayed increased for 60 min post intervention (microstate B for 5 min). Time coverage of microstate C was significant until and including 30 min after intervention. Frequency of occurence for microstate D was significantly decreased 5 min after exercise only. Significant transition from other microstates to C for whole follow up period. Results were corrected for multiple comparisons using Bonferroni
Wollseiffen et al. (2016a)	50 (46)	40.9 (11.1)	Five-arm, randomized	Intervention 1: 20 min, 70% of maximum heart rate, cycle ergometer, Intervention 2: 3 min maximum exhaustion boxing	Healthy	Fp1, Fp2, FPz, frequency analysis	Alpha-2 activity was significantly increased after boxing and biking in comparison with the usual break and no break, alpha-2 activity slightly increased after the massage chair intervention to the usual break and no break condition
Wollseiffen et al. (2016b)	11 (45.5)	36.5 (7)	Single group	6 h, at self-selected pace, running outdoors	Healthy ultra-marathoners	Fp1, Fp2, F3, Fz, F4, F7, F8, C3, C4, Cz, P3, Pz, P4, O1, Oz, O2, frequency analysis	Beta activity decreased after 6 h compared to pre in frontal areas
Woo et al. (2009)	16 (100)	21 (0.9)	Cross-over	15, 30, and 45 min, 60% VO_2_ max, treadmill	Healthy, non-regular exercisers	F3, F4, frequency analysis, frontal asymmetry	Frequency analysis: Increase in delta, theta (only right hemisphere) and alpha after 15 min of exercise. Asymmetry analysis: Higher frontal asymmetry scores after 30 min vs. rest, not significant for 15 and 45 min (delta, theta and alpha)
Woo et al. (2010)	16 (100)	21 (0.8)	Cross-over	30 min, graded (45, 60, and 75% of VO_2_ max), treadmill	Healthy, and had not exercised aerobically for the previous year	F3, F4, frequency analysis, frontal alpha asymmetry	Frequency analysis: Decreased left frontal power in all conditions compared to after rest. Asymmetry analysis: Increased frontal alpha asymmetry for all conditions compared to rest
Moraes et al. (2011)	29	Old age group: 70.4 (7) young age group: 25 (1.5)	Parallel group	20 min, 80% of age-predicted maximal heart rate, cycle ergometer	Healthy, moderately active	Fz, Cz, Pz,Oz, Fp1, Fp2, F3, F4, F7, F8, C3, C4, T3, T4, T5, T6, P3,P4, O1, O2, frequency analysis, LORETA	Significant increase in alpha and beta1 and decrease for beta2 across both groups. LORETA: Statistically significant increases post vs. pre in young for alpha (frontal), beta1 (anterior cingulate gyrus), beta2 (posterior cingulate gyrus). There were no significant between-group differences (pre-to-post). LORETA results were adjusted for multiple testing, but not power analysis and not for number of groups or frequency bands tested against each other
Hübner et al. (2018)	41 (Intervention: 53, Control: 52)	Intervention: 68.17 (3.18) [17] Control: 70.48 (2.75) [21]	Parallel group	20 min 60% max wattage, cycle ergometer	Healthy, physically active	Fp1, Fp2, F7, F3, Fz, F4, F8, FC5, FC3, FC1, FC2, FC4, FC6, T7, C3, Cz, C4, T8, CP5, CP3, CP1, CP2, CP4, CP6, P7, P3, Pz, P4, P8, O1, Oz, O2, frequency analysis	Beta power increased from before exercise intervention to after motor learning block. Time * group interaction was not significant. Bonferroni correction was applied
Vogt et al. (2010)	18 (44.4)	62.9 (5.3)	Single group	45–60 min self-selected pace outdoors walking.	Healthy	Fp1, Fp2, frequency analysis, assymmetry score	Right frontal alpha-1 and theta was higher than left
Dishman et al. (2010)	36 (Moderate intensity: 66, low intensity: 75 and controls: 83)	Moderate intensity: 23 (4.2), low intensity: 24 (4.7), control: 21 (2.4)	Two-arm, randomized, controlled	20 min, 3/week, for 6-weeks, graded, 75% or 40% VO_2_ max, cycle ergometer	Healthy	256-sensor Geodesics Sensor Net, frequency analysis	Higher activity in theta, alpha, low and high beta after low-intensity compared to control, alpha activity after low-intensity higher than after moderate-intensity. Otherwise, activity did not differ between groups
Gutmann et al. (2015)	10 (0)	22.7 (2.0)	Single group	30 min, 12 sessions, over the course of 4-weeks, 50% of peak power output ~65–75% HR max. Intensity increased by 5% each week, cycle ergometer (one-legged cycling)	Healthy, regular exercisers	Fp1, Fp2, F3, Fz, F4, T7, C3, Cz, C4, T8, P3, Pz, P4, O1, O2, individual alpha peak	Individual alpha peak frequency was increased immediately and 15 min after exhaustive exercise both before and after chronic intervention
Gutmann et al. (2018a)	10 (0)	22.7 (2.0)	Single group	Same as Gutmann et al. (2015)	Healthy	Same as Gutmann et al. (2015), frequency analysis, individual alpha peak based alpha band definition	Individual alpha peak shifted to a higher frequency as in Gutmann et al. (2015). Lower and upper alpha power shifted to higher power post vs. pre in exhaustive exercise group with individual alpha band definition
Kubitz and Landers (1993)	30 (60)	23.04 (3.62)	Randomized, controlled	40 min, 3/week, for 8-weeks, 60–85% of heart rate reserve, cycle ergometer	Healthy, non-regular exercisers	F3, F4, frequency analysis, alpha and beta laterality	No significant changes
Ludyga et al. (2017)	22 (Low cadence training: 27, High cadence training: 36)	27 (4)	Two-arm, randomized	4 h/week, for 4-weeks, individual heart rate targets (70–80% pulse at individual anaerobic threshold). High cadence and low cadence groups also engaged in four 60 min sessions of supervised cadence specific exercise weekly, outdoors and indoors cycling	Healthy cyclists	Fz, F3, F4, F7, F8, frequency analysis	No significant changes
Severtsen and Bruya (1986)	10 (100)	19–50 (range)	Two-arm, randomized	15–20 min per day at a self-selected intensity, for 7-weeks, instrument not specified	Healthy	Not stated, proportion of alpha and beta waves	No significant changes
Zilidou et al. (2018)	54 (Intervention: 95.5, Control: 77.3)	Intervention: 68.73 (4.73), control: 66 (5.51)	Randomized, controlled	60 min, 2/week for 24-weeks, traditional greek dance program	Healthy, sedentary	EASYCAP EEG cap, sLORETA, connectivity analyses, cortical synchronization analysis, cortical brain network analysis	Time * intervention interaction significant for small world value and characteristic path for 10.000 (small world),12.500 (small world) and 15.000 (characteristic path) edges respectively. Executive network betweenness centrality (BC) and within-module z-score (ZM) time * intervention interaction significant. Fronto-parietal network BC, ZM and participation coefficient (PC) time * interaction significant. Default mode network PC time * interaction significant as well as different between interventions. All networks PC time * interaction significant and different between interventions along with BC
**Persons with disability/disease** **<** **50 years, acute intervention**							
**References**	***N*** **(female percentage if stated)**	**Mean age (sd) for whole cohort if not stated otherwise**	**Study design**	**Intervention**	**Participants**	**EEG paradigm**	**Main results**
Brümmer et al. (2011a)	12 (Experiment 1: 33.3, Experiment 2: 0)	Experiment 1: 26.3 (3.8), experiment 2: 39 (7.9)	Experiment 1: Quasi-randomized cross-over, Experiment 2: Single-arm	Experiment 1: Treadmill (30 min), bicycle (30 min), arm crank (3 × 10 min) and isokinetic dynamometer (3 × 20 consecutive wrist flexions) at 50 and 80% of VO_2_ max and 50 and 80% of target intensity for isokinetic dynamometer; For experiment 2: Incremental arm crank test starting at 20 W and increasing with 20 W at each step for 5 min until volitional exhaustion	Experiment 1: Healthy recreational runners, Experiment 2: Patients with spinal cord injury group	Experiment 1: Fp1, Fp2, F3, F4, F7, F8, Fz, C3, C4, Cz, P3, P4, P7, P8, Pz, T7, T8, O1, O2; Experiment 2: Fp1, Fp2, F7, F3, Fz, F4, F8, FC5, FC1, FC2, FC6, T7, C3, Cz, C4, T8, TP9, CP5, CP1, CP2, CP6, TP10, P7, P3, Pz, P4, P8, PO9, O1, Oz, O2, PO10, sLORETA	Experiment 1: 50% intensity, alpha activity: Increase after treadmill, bicycle and arm crank in parietal, parietal and frontal areas respectively; 80% intensity, alpha activity: No significant differences, 50% intensity, beta activity: Increase after bicycle in parietal area; 80% intensity, beta activity: after treadmill, decrease frontal area. Experiment 2: Decreased frontal alpha activity. *T*-test statistics were corrected for multiple comparisons, but results were not corrected for number of groups or frequency bands
Vogt et al. (2012)	12 (0)	22.5 (9.87)	Single group	30 min, self-selected moderate pace, outdoors running	Persons with intellectual disability, relatively fit	FP1, FP2, F7, F3, Fz, F4, F8, FC5, FC1, FC2, FC6, T7, C3, Cz, C4, T8, TP9, CP5, CP1, CP2, CP6, TP10, P7, P3, Pz, P4, P8, PO9, O1, Oz, O2, PO10, LORETA	Decrease in cortical current density in rectal gyrus, orbital gyrus and Brodmann area 11. Contrast for post-cognitive task against pre-exercise showed a significant decrease of current density in medial frontal gyrus, but not immediately after exercise
Sato et al. (2017)	Wheelchair users: 11 (9), Healthy controls: 10 (10)	Wheelchair users: 46 (12.7) Healthy controls: 43 (11.1)	Parallel group	15 min, maximum intensity, wheelchair propulsion	Wheelchair users with spinal cord injury and tetra/paraplegia and healthy controls	Fp1, Fp2, AF3, AF4, F7, F3, Fz, F4, F8, FC5, FC1, FC2, FC6, T7, C3, Cz, C4, T8, CP5, CP1, CP2, CP6, P7, P3, Pz, P4, P8, PO3, PO4, O1, Oz, O2 divided into frontal (Fp1, Fp2, F3, Fz, F4), central (FC1, FC2, C3, Cz, C4), parietal (CP1, CP2, P3, P4, Pz) and occipital (O1, O2, Oz) regions of interest., peak alpha frequency	Peak alpha frequency changed to higher value for central region of interest
**Persons with disability/disease** **>** **50 years, acute intervention**							
**References**	***N*** **(female percentage if stated)**	**Mean age (sd) for whole cohort if not stated otherwise**	**Study design**	**Intervention**	**Participants**	**EEG paradigm**	**Main results**
Lattari et al. (2018)	10	Intervention: 36.4 (3.5), controls: 42 (8.4)	Randomized, controlled	Intervention: 12 sessions with 48–72 h interval between sessions consisting of 50–55% heart rate reserve, treadmill	Patients with anxiety disorder according to DSM IV	Fp1, Fp2, Fz, F3, F4, F7, F8, Cz, C3, C4, T3, T4,T5, T6, Pz, P3, P4, O1, O2, but only analyzed F3-F4, frontal alpha asymmetry	No significant changes
**Persons with disability/disease** **>** **50 years, acute intervention**							
**References**	***N*** **(female percentage if stated)**	**Mean age (sd) for whole cohort if not stated otherwise**	**Study design**	**Intervention**	**Participants**	**EEG paradigm**	**Main results**
Kamp and Troost (1978)	30	Intervention: 57(7.5), control 1: 20–30 (range) control 2: >50	Parallel group	Graded (till twice the resting heart rate or 160 bpm), cycle ergometer.	Cerebrovascular accident patients and healthy controls	12 electrodes (but only analyzed A2-O2, A1-O1, C4-P4, C3-P3), frequency analysis	Patients with cerebrovascular accidents showed a decrease in alpha frequency compared to normal individuals
**Persons with disability/disease** **>** **50 years, chronic intervention**							
**References**	***N*** **(female percentage if stated)**	**Mean age (sd) for whole cohort if not stated otherwise**	**Study design**	**Intervention**	**Participants**	**EEG paradigm**	**Main results**
Amjad et al. (2019)	40 (Intervention: 47, Control: 48)	Intervention: 58.23 (2.31), control: 59.56 (2.65)	Randomized, controlled	18 sessions (6-weeks) 20–40 min, 60–80% of maximum heart rate, stationary bicycle.	Patients with mild cognitive impairment (MMSE or MOCA <25 points)	AF3, F7, F3, FC5, T7, P7, O1, O2, P8, T8, FC6, F4, F8, AF4, relative frequency analysis, approximate entropy as a measure of complexity	Eyes closed: Decrease in delta and beta-1. Increase in alpha-2. Significant increase in approximate entropy. Eyes open: No significant differences for power or approximate entropy
Carvalho et al. (2015)	22 (Physiotherapy: 20, Active training: 28.6, Strength training: 44.4)	Physiotherapy: 64.8 (11.9), active training: 64.1 (9.9), strength training: 62.1 (11.7)	Three-arm, randomized	12-weeks, Physiotherapy: Calisthenics program, stretching, and gait training; Aerobic training: 60% of VO_2_ max or 70% of HR max 30 min treadmill; Strength training: exercises for large muscle groups using equipment for leg extensions, leg curls, leg presses, chest presses, and low row	Patients with Parkinson's disease	Fz, Fp1, Fp2, F3, F4, F7, F8, Cz, C3, C4, Pz, P3, P4, T3, T4, T5, T6, Oz, O1, O2, mean frequency	Aerobic and strength training groups had higher mean frequency compared with physiotherapy, but ANOVA showed no significant interaction for group * moment
**Persons with disability/disease** **<** **50 years, acute intervention**							
**References**	***N*** **(female percentage if stated)**	**Mean age (sd) for whole cohort if not stated otherwise**	**Study design**	**Intervention**	**Participants**	**EEG paradigm**	**Main results**
Deslandes et al. (2010)	20 (70)	71 (3)	Non-randomized, controlled	20 min 60% VO_2_ max, 2/week, unclear duration	Patients with major depressive disorder	Fz, Cz, Pz, Oz, Fp1, Fp2, F3, F4, F7, F8, C3, C4, T3, T4, T5, T6, P3, P4, O1, O2, frequency analysis, alpha asymmetry score	No significant changes
Silveira et al. (2010)	20 (90)	Intervention: 72.8 (5.1), control: 69.5 (3.7)	Non-randomized, controlled	20 min 60% VO_2_ max, 2/w, 6 months, treadmill	Patients with major depressive disorder	Fz, Fp1, Fp2, F3, F4, F7, F8, Cz, C3, C4, Pz, P3, P4, T3, T4, T5, T6, Oz, O1, O2, mean frequency	No significant changes
Styliadis et al. (2015)	70 (All groups: 64.3)	Long lasting memory training:71.21 (4.52), cognitive training: 70.42 (6.63), physical training: 72.71 (6.57), active control: 71.07 (4.38), passive control:67.64 (3.97)	Five-arm, non-randomized, controlled	8-weeks: Long lasting memory training (cognitive training, aerobics, strength, balance and flexibility): up to 10 h/w, Physical training (Physical component of long lasting memory training): up to 5 h/w, Cognitive training (Only cognitive part of long lasting memory training): 3 to 5 h/w, Active control (watched documentaries): up to 5 h/w	Patients with mild cognitive impairment according to Petersen criteria	EASYCAP EEG cap, eLORETA	Only LLM showed significant differences for the main study: decrease for delta, theta, beta 1 and beta 2 in the precuneus extending into the posterior cingulate cortex. Extra results for 14 MCI participants undergoing LLM treatment: significant decrease for delta, theta and beta-1 in precuneus /posterior cingulate cortex area. Beta-2 decrease in superior temporal gyrus. Multiple comparison adjustment was performed for LORETA *t*-test statistics, but not for number of frequency bands or groups tested against each other
Villafaina et al. (2019)	55(100)	Exercise: 52 (17), control: 54 (13)	Randomized, controlled	Exercise group: Exergame-based intervention, two 1 h sessions per week for 24-weeks. The exergames were comprised of: Warm-up, aerobic component, postural control and coordination games and walking training	Patients diagnosed with fibromyalgia according to the criteria of the American College of Rheumatology	Fz, Fp1, Fp2, F3, F4, F7, F8, Cz, C3, C4, T3, T4, T5, T6, Pz, P3, P4, O1 and O2, frequency analysis	Significant time*group interaction for increased beta-3 band power in frontal, parietal, temporal and occipital areas. Non-pre-specified subgroup analysis of long vs. short duration of symptoms: Significant increase in beta-3 in frontal and temporal area for exercise vs. control only for patients with short (<17 years) duration of symptoms. *P*-values were adjusted using the Benjamini-Hochberg procedure

*If multiple comparison adjustment in statistical analyses was performed in the included studies it is specified under main results*.

**Table 2:** EEG methods.

**References**	**EEG bands studied (with frequency spectrums)**	**EEG assessment interval(s) pre intervention**	**EEG assessment interval(s) post intervention**	**EEG analyzing software**	**Eyes closed assessment?**	**Eyes open assessment?**	**Collection time and epoch length (pre- and post-exercise if nothing stated for post)**
Kamp and Troost (1978)	Not stated	Not stated	Immediately afterwards	Not stated	Yes	Yes	Not stated for other than intervention group: 125 s, divided into three 12.5 s epochs
Villafaina et al. (2019)	Theta (4–7 Hz), alpha-1 (8–10 Hz), alpha-2 (11–12 Hz), beta-1 (13–18 Hz), beta-2 (19–21 Hz), and beta-3 (22–30 Hz)	1-week	1-week after last session	MATLAB	Yes	No	1 min, epoch length not stated
Hübner et al. (2018)	Beta (13–30 Hz)	Not stated	Not stated	Brain Vision Analyzer (Version 2.1, Brain Products GmbH, Gilching, Germany)	No	Yes	30 s divided into 2 s epochs
Vogt et al. (2010)	Delta (0.5–3.5 Hz), theta (3.5–7.5 Hz), alpha-1 (7.5–10.0 Hz), alpha-2 (10.0–12.5 Hz), beta-1 (12.5–18.0 Hz), beta-2 (18.0–35.0 Hz)	Immediately prior to	Immediately afterwards	Brain Vision Analyzer (Brain Products, Munich, Germany)	Yes	No	5 min divided into 4 s epochs
Zilidou et al. (2018)	Not stated	Not stated	Not stated	MATLAB Signal Processing Toolbox and EEGLAB. The Brainstorm software package.	Yes	No	Not stated, but divided into 2.048 s epochs
Silveira et al. (2010)	Mean frequency	Not stated	Not stated	MATLAB 5.3 (The Mathworks Inc., Natick, Mass., USA)	Yes	No	8 min, epoch length not stated
Deslandes et al. (2010)	Alpha (8–13 Hz)	Not stated	Not stated	EEGLAB	Yes	No	8 min divided into 4 s epochs
Amjad et al. (2019)	Delta (0.5–4 Hz), theta (4–8 Hz), alpha-1 (8–11 Hz), alpha-2 (11–14 Hz), beta-1 (14–25 Hz) and beta-2 (25–35 Hz)	Not stated	Not stated	MATLAB 2015	Yes	Yes	2 min, epoch length not stated
Styliadis et al. (2015)	Delta (2–4 Hz), theta (4–8 Hz), alpha (8–12 Hz), beta-1 (12–18 Hz), and beta-2 (18–30 Hz)	Not stated	Not stated	Not stated	Yes	No	5 min from which 15 4 s epochs were randomly extracted
Carvalho et al. (2015)	Mean frequency	Not stated	Not stated	MATLAB 5.3 (The Mathworks Inc., Natick, Mass., USA)	Yes	No	8 min, epoch length not stated
Moraes et al. (2011)	Delta (0.5–3.5 Hz), theta (4–7.5 Hz), alpha (8–12 Hz), beta-1 (13–18 Hz), beta-2 (18–30 Hz)	Not stated	15 min	ERP Acquisition	No	Yes	8 min, epoch length not stated
Lattari et al. (2018)	Alpha (8–14 Hz)	1 day before intervention	Not stated	NeuroSpectrum-5 (Medical Instruments, São Paulo, Brazil)	Not stated	Not stated	Not stated
Brümmer et al. (2011a)	Alpha (7.5–12.5 Hz) and beta (12.5–35 Hz)	Not stated	Experiment 1: 17.6 (SD:2.9) min afterwards; Experiment 2: Immediately afterwards	BrainVision Analyzer (Brain Products GmbH)	Experiment 1: Yes; Experiment 2: Not stated	No	5 min, epoch length not stated
Bailey et al. (2008)	Theta (4.50–7.99 Hz), alpha-1 (8.00–10.49 Hz), alpha-2 (10.50–12.99 Hz), beta-1 (13.00–17.99 Hz) and beta 2 (18.00- 30.00 Hz)	Not stated	Immediately afterwards and 10 min	Brain Vision Analyzer (Version 1.04, Brain Products GmH)	No	Yes	Pre: Two 1 min recordings each divided into 30 2 s epochs; post: 1 min divided into 30 2 s epochs
Bixby et al. (2001)	Alpha (8–13 Hz)	0, 5, and 10 min	8, 18, and 28 min	Neuroscan software (Neuroscan Labs, Neurosoft, Inc., version 4.0)	No	Yes	2 min divided into 1 s epochs
Boutcher and Landers (1988)	8, 10, and 12 Hz (taken as alpha total power)	21 min (5 s recordings, 2 min intervals)	21 min (5 s recordings, 2 min intervals). EEG data was divided into periods of 7 min for analysis	PIT spectral analysis program from BMDP statistical software	Yes	No	Every two min with a 5 s recording time for 21 min, epoch length not stated
Brümmer et al. (2011b)	Not stated	1 min	Immediately afterwards	Brain Vision Amplifier and RecView software (Brain Products GmbH, Munich, Germany)	Yes	No	1 min (20 s used for analysis; epoch length not stated)
Ciria et al. (2018)	All frequencies examined with no a priori assumptions of clusters.	Not stated	Not stated	EEGLAB and Fieldtrip MATLAB toolboxes	Yes	No	Collection time not stated, but divided into 1 s epochs
Fumoto et al. (2010)	Theta (4–8Hz), low-frequency alpha (8–10 Hz), high-frequency alpha (10–13 Hz), and beta (13–30 Hz)	1 min	Not stated	ATAMAP II (Kissei Comtec Co., Nagano, Japan)	No	Yes	1 min divided into 1.28 s epochs
Teixeira Guimaraes et al. (2014)	Delta, theta, alpha and beta activity (frequencies not specified)	5 min	15 min	sLORETA: KEY Institute for Brain-Mind Research (University Hospital of Psychiatry, Zurich, Switzerland; http://www.uzh.ch/Keyinst/NewLORETA/LORTA01.htm)	Not stated	Not stated	5 min, division not stated but 120 epochs were used per individual
Gutmann et al. (2018b)	Alpha (7–13 Hz)	Not stated	Experiment 1: Immediately after and divided into four groups with different post-intervention intervals: immediately after, 30, 60, and 90 min; Experiment 2: Immediately after and 20 min	Not stated	Yes	No	1 min divided into 4 s epochs
Hicks et al. (2018)	Alpha (8–13 Hz)	Not stated	6, 14, 22, and 30 min	EEGLAB, MATLAB	Yes	Yes	8 min divided into 2.048 s epochs
Hilty et al. (2011)	Alpha (7.25–12.5 Hz) and beta (12.5–35 Hz)	Not stated	Immediately afterwards	Brain Vision Analyzer 2.0 (Brain Products, Munich, Germany)	Yes	Yes	Three 30 s segments divided into 1 s epochs
Hottenrott et al. (2013)	Theta (4.5–7.5 Hz), alpha-1 (7.5–10 Hz), alpha-2 (10–12.5 Hz), beta-1 (12.5–18 Hz), beta-2 (18–32 Hz)	Not stated	Not stated	Brain Vision Analyzer 2.0 (Brain Products, Germany)	No	Yes	1 min divided into 4 s epochs
Kubitz and Mott (1996)	Alpha (7.8–12.5 Hz) and beta (14.1–29.7 Hz)	2 min prior to intervention	8 min	Not stated	Not stated	Not stated	Sixteen 1.28 s sweeps
Kubitz and Pothakos (1997)	Theta (4.7–7.7 Hz), alpha (7.8–12.5 Hz) and beta (14.1–29.7 Hz)	2 min prior	3 min	Not stated	Not stated	Not stated	Sixteen 1.28 s sweeps
Lattari et al. (2016)	Alpha (8–12 Hz)	Not stated	Not stated	MATLAB 5.3 (The Mathworks, Inc.)	Yes	No	8 min divided into 1 s epochs
Mechau et al. (1998)	Delta (1.25–4.5 Hz), theta (4.75–6.75 Hz), alpha-1 (7–9.5 Hz), alpha-2 (9.75–12.5 Hz), beta-1 (12.75–18.5 Hz), beta-2 (18.75–35 Hz)	2 min	0–1 min after each stage	CATEEM system (MediSyst GmbH, Linden, Germany)	Yes	No	2 min and divided into 4 s epochs
Mierau et al. (2009)	Alpha (7.5–12.5 Hz) and beta (12.5–35 Hz)	3 min	Immediately afterwards	Brain Vision Analyzer (Brain Products, Munich,Germany)	Yes	No	Pre: 2 min divided into 2 s epochs; post: 2 min divided into 4 s epochs
Moraes et al. (2007)	Alpha (8–13 Hz) and beta (14–20 Hz)	8 min	Immediately afterwards	ERP Acquisition (Delphi 5.0 TM, USA)	Yes	No	8 min, epoch length not stated
Mott et al. (1995)	Alpha (6–13 Hz), beta-1 (14–20 Hz), beta-2 (21–30 Hz)	2 min	Every 5 min in the last 2 min of each 5 min exercise period and finally 8 min after the last exercise/control session.	Not stated	Yes	No	Sixteen 1.28 s sweeps
Ohmatsu et al. (2014)	Delta (0.5–3.5 Hz), theta (3.5–7.5 Hz), alpha-1 (7.5–10 Hz), alpha-2 (10–12.5 Hz), beta-1 (12.5–18 Hz), and beta-2 (18–35 Hz)	Not stated	Not stated	EMSE Suite 5.4 (Source Signal Imaging, Inc., La Mesa, CA, USA)	Yes	No	3 min divided into 3 s epochs
Petruzzello and Landers (1994)	Alpha (7.8–12.7 Hz)	Not stated	After completion of a questionnaire 5, 10, 20 and 30 min after intervention	Computer Scope data acquisition software	No	Yes	65.53 s two times divided into 2.05 s epochs
Petruzzello and Tate (1997)	Alpha (8–12 Hz)	At least a day in advance	Immediately afterwards and at min 5, 10, 20 and 30	Not stated	Yes	No	Eight 1 min sweeps, epoch length not stated
Pineda and Adkisson (1961)	Alpha index (8–13 Hz)	15–30 min	2–3 min	By hand	Yes	Yes	Pre: 10 min, epoch length not relevant; post: 15 min
Schneider et al. (2009b)	Alpha (7.5–12.5 Hz) and beta (12.5–35 Hz)	Immediately before	2, 15, and 30 min	Brain Vision Analyzer (Brain Products, Munich, Germany)	Yes	No	5 min divided into 4 s epochs
Schneider et al. (2009a)	Delta (0.5–3.5 Hz), theta (3.5–7.5 Hz), alpha-1 (7.5–10.0 Hz), alpha-2 (10.0–12.5 Hz), beta-1 (12.5–18.0 Hz), beta-2 (18.0–35.0 Hz), gamma (35.0–100.0 Hz)	Immediately before	Immediately afterwards and 15 min	Brain Vision Analyzer (Brain Products, Munich, Germany)	Not stated	Not stated	5 min of which only the last 3 min were used and divided into 4 s epochs
Schneider et al. (2010a)	Delta (0.5–3.5 Hz), theta (3.5–7.5 Hz), alpha-1 (7.5–10 Hz), alpha-2 (10–12.5 Hz), beta-1 (12.5–18 Hz), beta-2 (18–35 Hz), and gamma (35–48 Hz)	Not stated	Immediately afterwards and 15 min	Brain Vision Analyzer (Brain Products, Munich, Germany)	Yes	No	5 min divided into 4 s epochs
Schneider et al. (2010b)	Delta (2–4 Hz)	Not stated	Not stated	Brain Vision Analyzer (Brain Products, Munich, Germany)	Not stated	Not stated	Not stated
Severtsen and Bruya (1986)	Not stated	Before 7-week program	End of program	By hand	Yes	Yes	15 min, epoch division not stated
Spring et al. (2017)	Delta (0.5–3.5 Hz), theta (3.5–7.5 Hz), alpha (7.5–12.5 Hz) and beta (12.5–35 Hz)	Not stated	1.5 (0.5) min after each intervention	Microstate: Cartool software by Denis Brunet (brainmapping.unige.ch/cartool), power: BrainVision Analyzer (Brain Products, Munich, Germany)	Yes	No	3 min divided into 4 s epochs
Spring et al. (2018)	Microstates	Not stated	5,15,30,45 and 60 min	Cartool software	Yes	No	5 min, epoch length not stated
Wollseiffen et al. (2016a)	Alpha-1 (7.5–10 Hz), alpha-2 (10–12.5 Hz), beta-1 (13–18 Hz) and beta-2 (18–35 Hz)	Not stated	Immediately afterwards	Brain Vision Analyser 2 (Brain Products GmbH)	Yes	No	3 min divided into 4 s epochs
Wollseiffen et al. (2016b)	Alpha (8–13 Hz) and beta (13–35 Hz)	Not stated	After each hour of running	Brain Vision Analyzer 2 (Brain Products, Gilching, Germany)	Yes	No	2 min divided into 4 s epochs
Woo et al. (2009)	Delta (1–4 Hz), theta (4–7 Hz), alpha (8–13 Hz), beta-1 (13–22 Hz) and beta-2 (23–30 Hz)	At least a week before first training session	20 min	MATLAB	No	Yes	10 min divided into 1 s epochs
Woo et al. (2010)	Alpha (8–13 Hz)	A day in advance	20 min	MATLAB	No	Yes	10 min divided into 1 s epochs
Dishman et al. (2010)	Theta (4–7 Hz), alpha (8–13 Hz), low beta (13–20 Hz), and high beta (20–30 Hz)	Not stated	6 min	EEGLAB 4.515 in MATLAB (Version 7.0, MathWorks, Natick, MA)	Yes	No	4 min divided into 10 s epochs
Gutmann et al. (2015)	Alpha peak (7–13 Hz)	Not stated	Immediately afterwards and 10 min	Not stated	Yes	No	2 min divided into 4 s epochs
Gutmann et al. (2018a)	In this study the authors analyzed the data from Gutmann et al. (2015) again, this time using a definition for alpha band of individual alpha peak −2.5 Hz +3 Hz.	Not stated	Immediately afterwards and 10 min	Not stated	Yes	No	2 min divided into 4 s epochs
Kubitz and Landers (1993)	Alpha (5–12 Hz) and beta (13–28 Hz)	Not stated	Not stated	DataPac II power spectral analysis software	No	Yes	Two 1 min periods, epoch length not stated
Ludyga et al. (2017)	Alpha (7.5–12.49 Hz) and beta (12.5–32 Hz)	Not stated	Before final exercise session	BrainVision Analyzer 2.0	Yes	No	1.5 min divided into five 2 s epochs
Devilbiss et al. (2019)	Delta (1–4 Hz), theta (4–8 Hz), alpha (8–12 Hz), beta (12–30 Hz), gamma (>30 Hz)	Not stated	~2.5 min	Not stated	Yes	Yes	2 min divided into 8 s epochs
Sato et al. (2017)	Alpha (7–14 Hz)	Not stated	10 min	EMSE Suite 5.4 (Source Signal Imaging Inc., La Mesa, CA, USA)	Yes	No	3 min divided into 2 s epochs
Vogt et al. (2012)	Not stated	Not stated	Immediately afterwards	Brain Vision Analyzer 2.0 (Gilching,Germany)	Yes	No	3 min divided into 4 s epochs

### Exercise Intervention Regimes and Post-intervention Analysis Time

The intervention methods in the included studies are reported in detail in Table 1 and post-intervention analysis intervals are reported in Table 2. A cycle ergometer was the most used exercise intervention instrument in acute interventions (*N* studies = 25), followed by treadmill (*N* = 9), track running (*N* = 3), outdoors running (*N* = 3), home-trainer (*N* = 2), arm crank (*N* = 2), walking (*N* = 1), wheelchair propulsion (*N* = 1). Most studies applied an acute intervention ≥30 min (*N* = 14). Other reported lengths of acute interventions were ≥20 min (*N* = 6), ≥15 min (*N* = 5), and one study reported applying the intervention for ≤ 10 min. Generally, studies used a marker for intervention intensity, which was often a percentage of maximal heart rate (MHR) or maximal oxygen consumption (VO_2_ max), while others reported using a graded intervention until volitional exhaustion or until a critical point (certain percentage of heart rate or VO_2_ max) was reached (*N* studies = 15) or used other markers. The reported VO_2_ max intensities at which the participants were expected to perform in the acute interventions were: 50–60% VO_2_ max (*N* = 5), 60–70% VO_2_ max (*N* = 2), 70–80% (*N* = 7), 80–90% (*N* = 4). The reported MHR intensities at which the participants were expected to perform in the acute interventions were: 60–70% MHR (*N* = 1), 70–80% MHR (*N* = 1), 80–90% (*N* = 3). The post-intervention analysis interval for both acute and chronic interventions was not stated in *N* = 16 studies. The post-intervention analysis interval was reported as being immediately afterwards in 17 studies, ≤ 5 min (*N* = 8), ≤ 10 min (*N* = 6), ≤ 15 min (*N* = 2), ≤ 20 min (*N* = 2), ≤ 25 min (*N* = 1).

The chronic interventions reported on differed to such a large degree that a meaningful summary of the interventions reported is best appreciated by studying Table 1, where chronic intervention studies can be found adjoined.

### Analytical Methods of Included Studies

The most frequently reported method of analysis was frequency analysis, whereby the power of frequency bands was studied (*N* studies = 32, see Tables 1, 3–6 for listings of methods of the included studies). Other derivatives of frequency analysis in the studies included coherence (the correlation between hemispheres for a specific frequency band), laterality (quantifying the lateralization of power) and mean frequency. Another method of analysis frequently reported on was low resolution electromagnetic tomography (LORETA). The studies using LORETA all applied a standard neuroanatomical atlas on a 3D head model. Also studied was microstates (Spring et al., 2017, 2018). Lastly, brain connectivity was considered in one study (Zilidou et al., 2018). Brain connectivity was examined by the authors by applying the LORETA method, and further examining sources of activity as nodes in graph theory based understandings of brain networks (Rossini et al., 2019).

**Table 3:** Summary of main findings of frequency analysis of post- vs. pre-exercise measurements.

	**δ**	**θ**	**α**	**β**	**γ**
Acute intervention	**Woo et al. (****2009****), Mechau et al. (****1998****), Bailey et al. (****2008****)** *Spring et al. (2017)*(intervention 1)** Ciria et al. (2018), Devilbiss et al. (2019), Schneider et al. (2009a), Spring et al. (2017) (intervention 2)	**Woo et al. (****2009****), Spring et al. (****2017****) (intervention 2), Mechau et al. (****1998****)** *Devilbiss et al. (2019), Fumoto et al. (2010)* Ciria et al. (2018), Hottenrott et al. (2013), Schneider et al. (2009a), Spring et al. (2017) (intervention 1)	**Moraes et al. (****2011****), Woo et al. (****2009****), Wollseiffen et al. (****2016a****) (boxing and bike), Spring et al. (****2017****) (intervention 2), Schneider et al. (****2009a****) (low intensity), Mechau et al. (****1998****), Hicks et al. (****2018****), Bailey et al. (****2008****), Bixby et al. (****2001****), Boutcher and Landers (****1988****), Fumoto et al. (****2010****)** *Kamp and Troost (1978), Woo et al. (2010)* Ciria et al. (2018), Devilbiss et al. (2019), Hilty et al. (2011), Hottenrott et al. (2013), Kubitz and Mott (1996), Mierau et al. (2009), Moraes et al. (2007), Petruzzello and Landers (1994), Petruzzello and Tate (1997), Schneider et al. (2009a) (preferred and high intensity), Spring et al. (2017) (intervention 1), Wollseiffen et al. (2016b), Gutmann et al. (2018a) (individual band definition)	**Moraes et al. (****2011****, beta-1), Spring et al. (****2017****) (intervention 2), Moraes et al. (****2007****), Mechau et al. (****1998****), Bailey et al. (****2008****)**, *Moraes et al. (2011, beta-2), Schneider et al. (2009a)(preferred and high intensity), Wollseiffen et al. (2016b)* Ciria et al. (2018), Devilbiss et al. (2019), Fumoto et al. (2010), Hottenrott et al. (2013), Kubitz and Mott (1996), Mierau et al. (2009), Schneider et al. (2009a) (low intensity), Spring et al. (2017) (intervention 1), Wollseiffen et al. (2016a) (boxing and bike), Woo et al. (2009)	Devilbiss et al. (2019), Schneider et al. (2009a)
Chronic intervention	*Amjad et al. (2019) (eyesclosed)* Amjad et al. (2019) (eyes open)	Amjad et al. (2019) (eyes closed), Amjad et al. (2019) (eyes open), Villafaina et al. (2019), Dishman et al. (2010) (low, high and moderate intensity)	**Amjad et al. (****2019****) (eyes closed, alpha-2)** Kubitz and Landers (1993), Ludyga et al. (2017), Amjad et al. (2019) (eyes closed, alpha-1), Amjad et al. (2019) (eyes open), Deslandes et al. (2010), Villafaina et al. (2019), Dishman et al. (2010) (low, high and moderate intensity)	**Villafaina et al. (****2019****) (beta-3)** *Amjad et al. (2019) (eyes closed, beta-1)* Kubitz and Landers (1993), Ludyga et al. (2017), Amjad et al. (2019) (eyes closed, beta-2), Amjad et al. (2019) (eyes open), Villafaina et al. (2019) (beta-1 and beta-2), Dishman et al. (2010) (low, high and moderate intensity)	

*Results are ordered in specific frequency bands and whether the intervention was acute (single bout) or chronic (≥2-weeks). Bold, italic and underlined or normal text of author (year) signifies: **statistically significant increase**, statistically significant decrease, no statistically significant results. δ, θ, α, β, and γ refer to the individual studies' frequency band definitions (see Table 2 for specifications of these) although any study's definition of alpha-1 and alpha-2 as well as beta-1 and beta-2 have been collapsed into alpha and beta, respectively, in this table for simplification in cases where the direction of results for the sub-specified bands are the same. Studies that carried out multiple experiments with different exercise intensities/instruments or analysis methods are reported here for each intensity/instrument/method if results differed between intensities/instruments/methods. The intensity/intensities/instrument(s)/method(s) that gave the reported direction of change is indicated in parentheses. For further elaboration of results and EEG methods see Tables 1, 2, respectively. Studies that tested multiple time points post exercise against pre are only reported once if the study reported any significant findings and only for changes present in the first time point tested post vs. pre exercise*.

### Frequency Analysis

**Figure 2:**
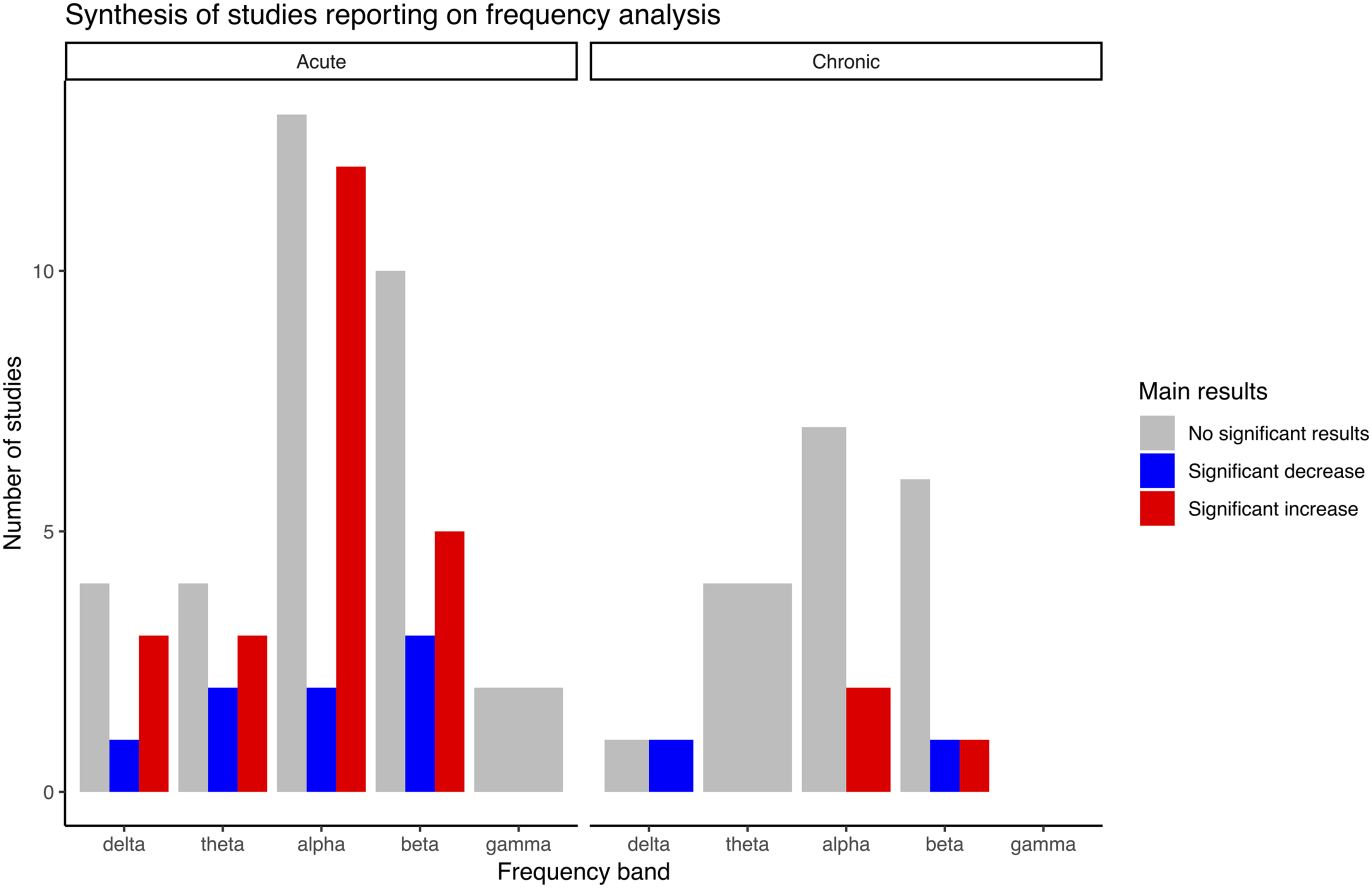
Main results synthesis bar plot of studies reporting on frequency analysis.

Table 3 summarizes the main findings of studies using frequency analysis, while Table 4 reports the findings of studies from which the outcomes are derived from frequency analysis (asymmetry, coherence, peak shifts, laterality, wave proportion, and mean frequency). Figure 2 summarizes results reported in Table 3, for visual simplification. In total, 32 studies carried out a frequency analysis and within these studies the effects of exercise intervention on alpha and beta band activity were overrepresented (Table 3). Definitions for these bands differed between studies (Table 2). In total, 21 studies reported on derivatives of frequency analysis. Some studies reported on both frequency analysis and derivatives and LORETA, which means that the summed total number of studies reported here for each category is larger than the total number of studies included in the present review.

**Table 4:** A summary of main findings from frequency analysis derivatives of post- vs. pre-exercise measurements.

	**δ/θ/α/β asymmetry**	**α/β coherence**	**α peak shift**	**α/β laterality**	**α/β wave proportion**	**Mean frequency**
Acute interventions	**Ohmatsu et al. (****2014****) (α-1)↑** Woo et al. (2010) (α)↑ Lattari et al. (2016) (α) Petruzzello and Tate (1997) (α) Schneider et al. (2009a) (δ/θ/α/β) Woo et al. (2009) (δ/θ/α/β) Hicks et al. (2018) (α)	**Mott et al. (****1995****)↑** **(α)** Mott et al. (1995) (β)	**Gutmann et al. (****2015****) (exhaustive exercise)** **↑** **Gutmann et al. (****2018b****) (graded and high)↑** Gutmann et al. (2018b) (moderate and low), Gutmann et al. (2015) (steady state exercise)			
*Chronic interventions*	Lattari et al. (2018) (α) Deslandes et al. (2010) (α)		Gutmann et al. (2015)	Kubitz and Landers (1993)	Severtsen and Bruya (1986)	Carvalho et al. (2015), Silveira et al. (2010)

**Significant change**, non-significant change. ↑↓ direction of change if applicable. δ, θ,α, and β refer to the individual studies' frequency band definitions (see Table 2 for specifications of these). Studies that carried out multiple experiments with different exercise intensities/asymmetry frequencies are reported here for each intensity/asymmetry frequency if results differed between intensities. The intensity that gave the reported direction of change is indicated in parentheses. For further elaboration of results and EEG methods see Tables 1, 2, respectively. *Includes cortical synchronization analysis and cortical network analysis.

#### Acute Interventions

In total, 25 studies investigated frequency analysis in acute intervention studies. Several studies found significant increases in power within the delta, theta, alpha and beta band, and although there was a quantitative overweight of studies reporting increases, there were also studies that reported significant decreases for all the same bands and a high number of studies reported no significant changes (Table 3). No significant changes were reported within the gamma band (Schneider et al., 2009a; Devilbiss et al., 2019). The results by Devilbiss were adjusted for multiple comparisons.

##### Derivatives of frequency analysis

Table 4 gives the results of studies on derivatives of frequency analysis in acute interventions with hypothesis tests (*N* = 10). Most of these studies investigated asymmetry (*N* = 7). Two studies (Woo et al., 2010; Ohmatsu et al., 2014) reported significant increases in alpha asymmetry, while five studies reported no significant changes. Within the studies reporting no significant changes, multiple frequencies were investigated (delta, theta, alpha and beta) in two studies (Schneider et al., 2009a; Woo et al., 2009). An alpha peak shift was observed in two studies by the same group (Gutmann et al., 2015, 2018b), both only showed in high intensity settings.

#### Chronic Interventions

In total, seven studies reported on frequency analysis results in chronic exercise interventions (Tables 1, 3). Significant increases of alpha-2 (Amjad et al., 2019) and beta-3 bands (Villafaina et al., 2019) were reported, while significant decreases were found for delta (Amjad et al., 2019), and beta-1 (Amjad et al., 2019). Results by Villafaina et al. were adjusted for multiple comparisons. There were reports of no significant changes within all frequencies with an overweight of studies done on alpha and beta bands. No significant findings were reported within the theta band.

##### Derivatives of frequency analysis

No studies reported significant changes within derivatives of frequency analysis for chronic exercise interventions (Table 4) (*N* = 7). The investigated derivatives of power spectrum analyses were asymmetry, alpha peak shift, laterality, wave proportion, and mean frequency.

### LORETA

In total, 12 studies reported using LORETA as an analytic method for resting state EEG measurements. Table 5 gives the summarized statistically significant results of these studies.

**Table 5:** A summary of main findings from studies using LORETA analysis of post- vs. pre-exercise measurements.

	**δ**		**θ**		**α**		**β**		
Acute intervention	Frontal: **Schneider et al. (****2010b**) (high intensity)^*^, Schneider et al. (**2010a****)**	Parietal:			**Schneider et al. (****2010a****) (alpha-1), Brümmer et al. (****2011a****) (Experiment 1, 50% intensity, arm crank), Moraes et al. (****2011****) (young)^†^, Schneider et al. (****2009b****) (arm crank, treadmill), Hilty et al. (****2011****)** *Brümmer et al. (2011a)(Experiment 2), Ohmatsu et al. (2014)^††^*	**Brümmer et al. (****2011a****) (Experiment 1, 50% intensity, treadmill and bicycle), Schneider et al. (****2009b****) (bike)** Brümmer et al. (2011b)^**^	**Moraes et al. (****2011****) (young)^‡^, Hilty et al. (****2011****)**	**Brümmer et al. (****2011a****) (Experiment 1, 50% intensity, bike), Moraes et al. (****2011****) (young)^§^, Schneider et al. (****2009b****) (arm crank, treadmill)** Brümmer et al. (2011a)(Experiment 1, 80% intensity, treadmill), Brümmer et al. (2011b)^**^	
	Temporal:	Occipital:	**Schneider et al. (****2010a****)**		**Teixeira Guimaraes et al. (****2014****) (alpha-2) (maximal effort)**		**Teixeira Guimaraes et al. (****2014****) (beta-2) (maximal effort)**	**Teixeira Guimaraes et al. (****2014****) (beta-2) (maximal effort)**	
Chronic intervention	Frontal:	Parietal: *Styliadis et al. (2015)(Longlastingmemorygroup)*		*Styliadis et al. (2015)(Longlastingmemorygroup)*				*Styliadis et al. (2015)(Long lastingmemory group)*	
	Temporal:	Occipital:							

*Results are ordered in specific frequency bands and whether the intervention was acute (single bout) or chronic (>=2 weeks) along with bilaterally defined brain lobal anatomical localization (Top left to bottom right in right-left reading direction for each frequency/intervention type-defined square: frontal, parietal, temporal, occipital; see upper-left square for this schematic presentation). Bold, italic and underlined or normal text of author (year) signifies: **statistically significant increase**, statistically significant decrease. δ, θ, α, β, and γ refer to the individual studies' frequency band definitions (see Table 2 for specifications of these) although any study's definition of alpha-1 and alpha-2 as well as beta-1 and beta-2 have been collapsed into alpha and beta, respectively, in this table for simplification in cases where the direction of results for the sub-specified bands are the same. Studies that carried out multiple experiments with different exercise intensities/instruments or analysis methods are reported here for each intensity/instrument/method if results differed between intensities/instruments/methods. The intensity/intensities/instrument(s)/method(s) that gave the reported direction of change is indicated in parentheses. Studies that tested multiple time points post exercise against pre are only reported once if the study reported any significant findings and only for changes present in the first time point tested post vs. pre exercise*.

** *Analyzed 6–49 Hz spectrum total power*.

* *Brodmann area (BA) 9 and 32*.

† *BA 24*.

‡ *BA 33*.

§ *BA 23*.

†† *BA 32*.

#### Acute Interventions

Ten studies investigated LORETA in conjunction with an acute exercise intervention. There were significant findings within all bands with an overweight of significant increases in the alpha and beta bands, which for alpha were found in all but the occipital lobe, whereas beta increases were found in all lobes (see Tables 1, 5). Significant decreases were found within the alpha band (frontal and parietal lobe) (Brümmer et al., 2011a,b; Ohmatsu et al., 2014) and beta band (parietal lobe) (Brümmer et al., 2011a,b). Changes were often localized to small brain areas (see Table 5), with inconsistent findings across studies with regards to anatomical localization of reported changes.

#### Chronic Interventions

Two studies applied LORETA to their data (Styliadis et al., 2015; Zilidou et al., 2018) in chronic intervention studies. Of these, one study reported significant findings. These were decreases in delta, theta and beta bands (all located to parietal lobe) (Styliadis et al., 2015).

### Other Methods

Table 6 gives the results of studies investigating aspects of EEG analysis other than frequency analysis and LORETA.

**Table 6:** A summary of main findings of other methods of analysis of post- vs. pre-exercise measurements.

	Brain connectivity^*^	**Microstate analysis**	**Mean lagged synchronization**	**Approximate entropy**
Acute interventions		**Spring et al. (****2017****) (microstate C)** **Spring et al. (****2018****) (microstate B and C)**	Hilty et al. (2011)	
Chronic interventions	**Zilidou et al. (****2018****)**			**Amjad et al. (****2019****) (eyes closed)↑** Amjad et al. (2019) (eyes open)

***Significant change**, non-significant change. ↑↓ Direction of change if applicable. δ, θ,α, and β refer to the individual studies' frequency band definitions (see Table 2 for specifications of these). Studies that carried out multiple experiments with different exercise intensities/asymmetry frequencies are reported here for each intensity/asymmetry frequency if results differed between intensities. The intensity that gave the reported direction of change is indicated in parentheses. For further elaboration of results and EEG methods see Tables 1, 2, respectively*.

#### Acute Interventions

In total, three studies reported findings from investigations involving microstate analysis and mean lagged synchronization. Studies on microstates used a definition of four distinct EEG microstates called A, B, C, and D, generally accepted in the literature (Lehmann et al., 2009). Significant findings were reported for microstate analysis, showing that the global variance explained (Spring et al., 2017), mean duration of Spring et al. (2017, 2018), and time coverage (Spring et al., 2017, 2018) for microstate C and mean duration of microstate B (Spring et al., 2018) were all increased after exercise.

#### Chronic Interventions

In total, two studies reported on investigations involving brain connectivity and approximate entropy. The authors of one study considered graph theory based brain networks before and after a 24-week traditional Greek dance program (Zilidou et al., 2018). Zilidou et al. modeled the cortex with 20,000 fixed dipoles which were grouped into 512 cortical regions of interest (ROIs) Significant time × intervention group interactions were found when measuring how much participants brain behaved as a small world, which essentially is an expression of local information processing and brain connectivity, when the threshold of connections between regions-of-interest (ROIs) were set at 10,000 and 12,500, which is an arbitrary measure used to model the network system. Several measures pertaining to graph theory were investigated including the shortest path length between two ROIs, which had a significant time × intervention group interaction, when a connection threshold of 15,000 was applied. Further, measurements of information flow through particular ROIs, the tendency of specific ROIs to behave as hubs for information, the connection distributions and connection strengths were similarly significantly changed within different brain networks. In another study, it was found that the approximate entropy, which is a measure of how complex a network is, was found to be increased in the eyes closed condition after a chronic exercise intervention (Amjad et al., 2019). We refer to the original studies for in-depth explanations of specific measures investigated in these included studies.

### Risk of Bias

**Figure 3:**
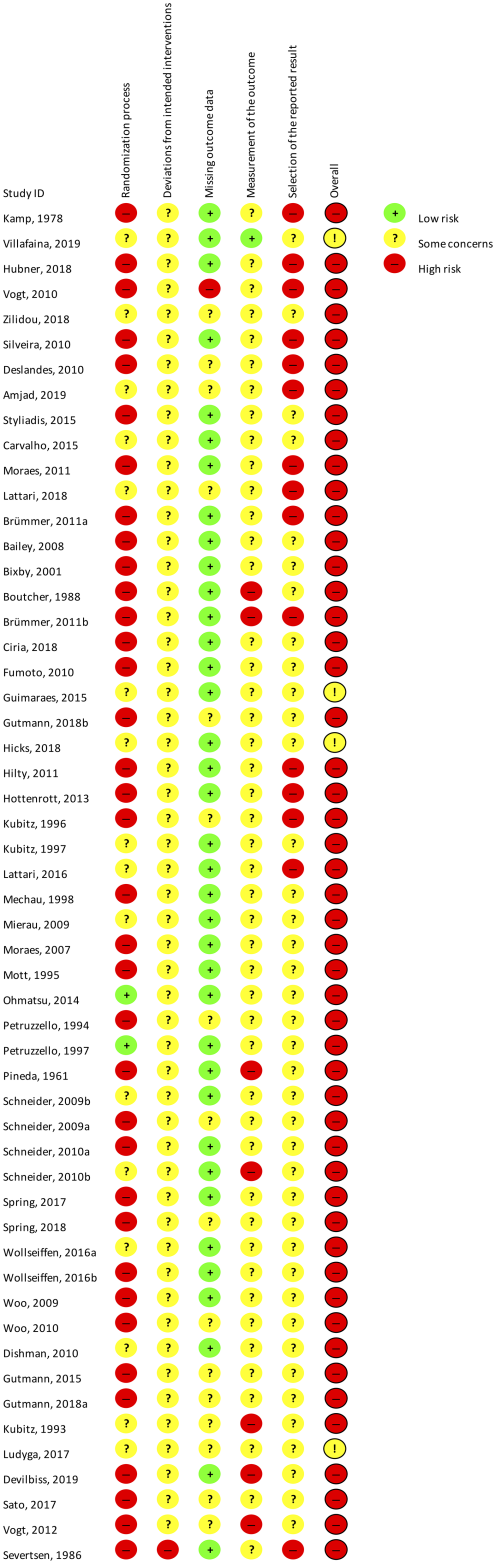
Cochrane risk of bias assessment of all included studies.

**Figure 4:**
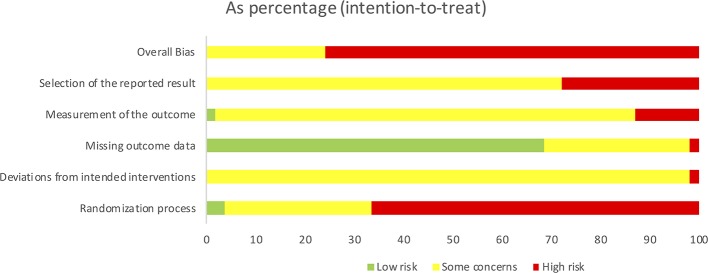
Summarized Cochrane risk of bias assessment graph for all included studies.

The summarized results of the risk of bias assessment for each study are shown in Figure 3. Figure 4 gives the percentages of individual assessments of specific domains addressed in the risk-of-bias tool. An overweight of studies were rated as having an overall high risk of bias (*N* = 41), whereas the rest of the included studies were rated as having some concerns overall (*N* = 13). No studies were rated as having low risk of bias within the domain pertaining to selection of the reported result. Overall bias ratings were mainly influenced by the domains “Selection of the reported result,” “Randomization process” (see Figure 4).

## Discussion

### Summary of Main Findings

We carried out a systematic review of studies investigating the effects of exercise interventions on resting state EEG/MEG. After screening, 54 studies were included in the final qualitative synthesis. Results of this synthesis showed that studies in general were small, and carried out in healthy, young individuals applying an acute exercise intervention. The most often used methodology for EEG analysis was frequency analysis and its derivatives. The results of summarized frequency analysis findings for the acute and chronic effects of exercise were inconsistent for most frequency bands. There was some indication of a delta band activity decrease after chronic interventions and a null effect in the theta and gamma band, and an alpha peak shift after acute exercise with high intensity. Anatomical source localization studies using LORETA reported few localized increases within the delta (frontal) and theta band (temporal) in acute exercise and decreases within delta and theta (both parietal) after chronic exercise interventions. Moreover, more widespread alpha and beta activity increases were shown after acute exercise, although decreases in some of these areas were also reported demonstrating the inconsistency across studies. Other methods of analyzing EEG data revealed significant changes in brain connectivity after a chronic dance intervention. Replicated changes of microstate C after acute exercise, which were adjusted for multiple comparisons, were also found, although not independently validated. In all but 13 studies, where there were some concerns regarding risk of bias, there was an overall high risk of bias indicating a low quality of studies. No studies were found reporting on MEG in exercise interventions.

In the present review, we aimed to include all available data on exercise interventions and resting state EEG measurements. Previous reviews on the subject have had other focus areas, such as the ability of EEG to predict mood states in exercise interventions (Lattari et al., 2014), and one review had the inclusion criteria that the included studies could be assessed by quantitative analysis (Crabbe and Dishman, 2004). Our conclusions are somewhat in contrast with what Crabbe and Dishman reported, which is the review closest in aim to ours. They found widespread increase of activity in all frequency bands after exercise. Unexpectedly, we did not find this to be the case when we considered all available data. Differences in the selection process of included studies might have influenced this discordance in conclusions. Moreover, several studies have subsequently been published, which we were able to include, and which may have influenced our finding. We also included studies on patient populations, and it could be argued that these results may have biased our conclusions, e.g., that different brain pathologies interfere with the effects of exercise. However, the only study showing a decrease in delta activity in chronic interventions was done on patients with mild cognitive impairment (Amjad et al., 2019). This would contradict such a notion, although the generalizability of this finding is limited. As such, only few of the included studies were performed in patients, and it is unlikely that the results from these studies impaired the conclusions that could be drawn from our synthesis. Our rationale for including patient populations was the fact that exercise has been proposed as a possible therapeutic option in some diseases (Pedersen and Saltin, 2015), and as such it could interesting to see if an effect was evident in patient populations.

Within most frequency bands, findings were inconsistent for studies reporting on frequency analysis. A single study reported a decrease in delta activity in chronic interventions in an eyes closed condition in patients with mild cognitive impairment (Amjad et al., 2019), and it was not replicated by other groups. A null effect seemed present in acute interventions for gamma activity, and in chronic interventions for theta activity, while for the rest of frequency bands, both in acute and chronic interventions, the results were too inconsistent to draw any conclusions regarding the direction of change. The review by Crabbe and Dishman did not look at gamma activity and thus we are the first to synthesize results on this frequency band in exercise interventions. Gamma activity is related to working memory (Uhlhaas et al., 2011) and since exercise has been shown to improve memory (Smith et al., 2010) a connection between exercise and gamma activity might be probable. There were two studies investigating this band (Schneider et al., 2009a; Devilbiss et al., 2019). The study by Devilbiss et al. recorded EEG from a single frontally placed electrode, which means that changes in gamma activity will have been missed if this occurred in other brain areas than in frontal areas. The results by Devilbiss et al. were adjusted for multiple comparisons. The study by Schneider et al. was done in 24 participants. Thus, a true effect could have been missed due to low statistical power. It was also very rare that studies reporting on frequency analysis adjusted for multiple comparisons, which means that results in effect could be due to randomness, as adequate adjustment would include the number of frequency bands, number of groups and the number of time points tested against each other, in general. Further research is needed in the area of gamma activity and exercise as few groups looked into this aspect.

A considerable amount of studies reported using LORETA as an EEG analysis method. Generally, studies reporting on LORETA showed great heterogeneity with regards to which areas were affected by exercise, and while several studies found increases or decreases confined to individual Brodmann-defined areas, no widespread consistent intra-lobal increase/decrease was reproducibly shown. A single region, the dorsal anterior cingulate area, was shown to have increases in delta activity (Schneider et al., 2010b) and concurrent decrease in alpha activity (Ohmatsu et al., 2014) in acute intervention studies. The cingulate region is involved in executive control, emotion and spatial memory among other brain functions (Bubb et al., 2018), and delta oscillations are also implicated in memory tasks (Harmony, 2013), which in turn can be modulated by exercise (Smith et al., 2010) making a connection possible, but must at present remain speculative. A limiting factor in both studies is the number of electrodes used to localize this activity. A recent review, commissioned by International Federation of Clinical Neurophysiology (IFCN), states that a minimum of 48–64 (and upwards of 128–256) electrodes might be warranted to achieve this approximation with a high enough degree of certainty (Babiloni et al., 2019). Nonetheless, application of LORETA analysis methods represents an interesting area to elucidate anatomical areas affected by exercise. As shown by the few studies identified by this review, it should be explored further with more strict methodology to confirm the exact anatomical localization of these effects.

We chose to include studies on different patient groups in the present review, with the exclusion of sleep disorders and epilepsy, which are diseases where a different EEG/MEG approach might be warranted than the ones generally reported here. Generally, few studies were carried out in diseased individuals with our inclusion criteria and there was great heterogeneity in the diseases studied. A few studies deserve some special attention, since they focused on the same disease, namely patients with spinal cord injuries (Brümmer et al., 2011a; Sato et al., 2017), and thus offer some unique insights. The study by Brümmer et al. involved participants who were semi-professional hand-cycling athletes and were included as a validation group to confirm specific exercise preferences related to frontal activity, whereas Sato et al. studied a less selected cohort of patients with spinal cord injury as their primary group and wished to investigate this group of patients specifically. Both interventions applied to this group consisted of aerobic arm exercises, namely wheelchair propulsion (Sato et al., 2017) and arm crank exercise (Brümmer et al., 2011a). Both studies reported on the alpha freqeuency, where Brümmer et al. found decreased frontal alpha activity, and Sato et al. found a shift to a higher alpha peak frequency, which is also found in healthy individuals (Gutmann et al., 2015) in chronic interventions. This could indicate that the effect on the alpha peak shift is preserved in patients with spinal cord injury.

The present review also identified studies using methods other than freqeuncy analysis and LORETA to analyze the EEG. An area that seems promising is the study of brain connectivity, which was studied by one group, investigating whether a 24-week intervention consisting of Greek traditional dancing had an effect on this aspect of brain function in healthy individuals (Zilidou et al., 2018). The study used LORETA based estimations of current source density and then applied graph theory based understandings of brain function assigning nodes to specific clusters of EEG activity. This enabled the group to estimate the network properties of the brain and the changes that occur when a chronic complex exercise intervention was applied in senior citizens. Within this paradigm, the group found changes in network connectivity when applying a threshold of 10,000 and 12,500 nodes in the small world property which is a measurement of how the network is connected (see for example Watts and Strogatz, 1998 for an introduction to graph theory). The group showed similarly significant changes in several areas of brain connectivity. No consensus currently exist as to the node threshold that should be applied (Rossini et al., 2019) and thus the results need to replicated to confirm these findings with the presently applied thresholds.

Many studies reported on the effects of exercise on asymmetry, a quantitative measure of the incongruence between hemispheric oscillatory activity. Results were generally inconsistent regarding this aspect, both for acute and chronic interventions, with an overweight of studies rated with an overall high risk of bias and many studies reporting a null effect (Petruzzello and Tate, 1997; Schneider et al., 2009a; Woo et al., 2009; Deslandes et al., 2010; Lattari et al., 2016, 2018; Hicks et al., 2018). Several theories have been proposed as to the nature and meaning of differential hemispheric activation, the most prominent being that lateralization of brain activity is specifically connected with affective states (Davidson, 1992), which is also the hypothesis that many of these studies build upon in their investigations into exercise. In line with this assumption the participants' state of mind was often investigated with mood state questionnaires before and after exercise. If the idea of affective states being associated with specific mood states holds true, then it should follow that if exercise somehow modulated the mood states of participants an effect could then be measured using asymmetry as an indicator of this change. Our synthesis seems to point to a null effect of exercise on asymmetry in all frequency bands, and even though this is unexpected, since a growing body of literature points to a mood altering effect of exercise (Mikkelsen et al., 2017), it could be due to non-validity of the underlying hypothesis of the connection between asymmetry and mood states or simply that the exercise intervention applied did not result in such a change in mood, a focus area which we did not explore in the present review.

No studies reported on MEG in exercise interventions and it can only be speculated as to why we were unable to retrieve studies reporting on this aspect. Generally, MEG is expensive to undertake and as such may not always be readily available to researchers. It could be interesting to see if results found using EEG could be replicated and build upon using MEG, since MEG should also capture deeper electromagnetic sources, that are not reachable by EEG. This is an area, where future research is needed.

### Limitations

We acknowledge the limitations of the present review. The employment of a wider search method with more databases included could potentially have revealed additional relevant records although our strategy was among some of the highest rated combinations in a recent review of optimal databases for literature searches (Bramer et al., 2017). We chose to include studies on populations with different diseases in our synthesis, which could have biased our results. Studies reporting on diseased participants were few and in general no discernable trends in the findings convincingly indicated a different response to those studies reporting on healthy individuals. We employed a qualitative synthesis of results. The reason for this choice of synthesis was based on the a priori assumption of large heterogeneity in studies. It could be argued that a quantitative synthesis should have been carried out, but the trade-off is the exclusion of studies not conforming to such an analysis, which we believe leaves out important data. We did not consider relative power contra total power nor did we analyze eyes-open vs. eyes-closed conditions, which might have affected the interpretation of results. Further, we excluded studies involving an event-related potential as an outcome and studies involving sleep EEG. While these are valid areas of research, where knowledge seems limited, it is beyond the scope of the present review. We also must refer to reviews commissioned by the IFCN for detailed descriptions of analytical methods as an in-depth guide for researchers is beyond the scope of this review (Babiloni et al., 2019; Rossini et al., 2019).

## Conclusions

We conducted a comprehensive systematic review within a validated combination of science databases on studies using EEG as an outcome measure in exercise interventions. Our synthesis showed that the included studies were inadequately powered to assess the impact on EEG signal by exercise interventions. Further methodological issues leading to low quality of the identified studies, limits the conclusions which may be drawn. The most often reported methods of analysis were frequency analysis and LORETA. The results when assessed for all studies were inconclusive regarding any changes observed in a pre- and post-exercise comparison and the significance that could be attached to any statistically significant results was severely impaired due to a large proportion of studies being rated with an overall high risk of bias as well as due to missing adjustment for multiple comparisons. There are indications that short bouts of exercise are associated with changes in EEG microstates, although independent validation studies should be carried out in larger populations. No studies were retrieved by our searches were MEG was the outcome, which means research in this area is lacking. EEG remains an interesting methodology to examine underlying effects of exercise on brain functions. Future studies should investigate larger populations and adhere to stricter methodology as well as report and carry out statistically meaningful power estimations prior to conducting experimental research with EEG as an outcome measure.

## Author Contributions

KF developed the idea for the review. MG crafted and conducted the bibliographic searches. MG and KF screened articles for inclusion and extracted data and assessed risk of bias. MG drafted the manuscript. KF, GW, and SH revised critically for important intellectual content and approved the manuscript.

## Conflict of Interest

The authors declare that the research was conducted in the absence of any commercial or financial relationships that could be construed as a potential conflict of interest.
