# Supplementary material for: Resting State EEG in Exercise Intervention Studies: A Systematic Review of Effects and Methods
Source: Front Hum Neurosci. 2020 May 7;14:155. doi: 10.3389/fnhum.2020.00155 (PMC7232557; doi:10.3389/fnhum.2020.00155)
Supplement: Supplementary file 1 [file Table_1.DOCX]

## Search strategy

For PubMed:

1. “exercise” [MeSh]
2. “exercise”
3. “electroencephalography” [MeSh]
4. “magnetoencephalography” [MeSh]
5. EEG
6. 1 OR 2
7. 3 OR 4 OR 5
8. 6 AND 7

For EMBASE

1. electroencephalography/
2. magnetoencephalography/
3. eeg.mp. [mp=title, abstract, heading word, drug trade name, original title, device manufacturer, drug manufacturer, device trade name, keyword, floating subheading word, candidate term word]
4. exercise/
5. 1 or 2 or 3
6. 4 and 5

For Web of Science:

| # 6 | [**823**](http://apps.webofknowledge.com.ep.fjernadgang.kb.dk/summary.do?product=WOS&doc=1&qid=6&SID=D2ffI7nuzVvNS9FiYSC&search_mode=CombineSearches&update_back2search_link_param=yes) | #5 AND #3  *Indexes=SCI-EXPANDED, SSCI, A&HCI, CPCI-S, CPCI-SSH, BKCI-S, BKCI-SSH, ESCI, CCR-EXPANDED, IC Timespan=All years* | [Edit](http://apps.webofknowledge.com.ep.fjernadgang.kb.dk/WOS_AdvancedSearch_input.do?product=WOS&SID=D2ffI7nuzVvNS9FiYSC&search_mode=AdvancedSearch&replaceSetId=6&editState=init) |  |  |
| --- | --- | --- | --- | --- | --- |
|  | | | | | |
| # 5 | [**114,190**](http://apps.webofknowledge.com.ep.fjernadgang.kb.dk/summary.do?product=WOS&doc=1&qid=5&SID=D2ffI7nuzVvNS9FiYSC&search_mode=CombineSearches&update_back2search_link_param=yes) | #4 OR #2 OR #1  *Indexes=SCI-EXPANDED, SSCI, A&HCI, CPCI-S, CPCI-SSH, BKCI-S, BKCI-SSH, ESCI, CCR-EXPANDED, IC Timespan=All years* | [Edit](http://apps.webofknowledge.com.ep.fjernadgang.kb.dk/WOS_AdvancedSearch_input.do?product=WOS&SID=D2ffI7nuzVvNS9FiYSC&search_mode=AdvancedSearch&replaceSetId=5&editState=init) |  |  |
|  | | | | | |
| # 4 | [**102,663**](http://apps.webofknowledge.com.ep.fjernadgang.kb.dk/summary.do?product=WOS&doc=1&qid=4&SID=D2ffI7nuzVvNS9FiYSC&search_mode=AdvancedSearch&update_back2search_link_param=yes) | TS=(EEG)  *Indexes=SCI-EXPANDED, SSCI, A&HCI, CPCI-S, CPCI-SSH, BKCI-S, BKCI-SSH, ESCI, CCR-EXPANDED, IC Timespan=All years* | [Edit](http://apps.webofknowledge.com.ep.fjernadgang.kb.dk/WOS_AdvancedSearch_input.do?product=WOS&SID=D2ffI7nuzVvNS9FiYSC&search_mode=AdvancedSearch&replaceSetId=4&editState=init) |  |  |
|  | | | | | |
| # 3 | [**426,758**](http://apps.webofknowledge.com.ep.fjernadgang.kb.dk/summary.do?product=WOS&doc=1&qid=3&SID=D2ffI7nuzVvNS9FiYSC&search_mode=AdvancedSearch&update_back2search_link_param=yes) | TS=(exercise)  *Indexes=SCI-EXPANDED, SSCI, A&HCI, CPCI-S, CPCI-SSH, BKCI-S, BKCI-SSH, ESCI, CCR-EXPANDED, IC Timespan=All years* | [Edit](http://apps.webofknowledge.com.ep.fjernadgang.kb.dk/WOS_AdvancedSearch_input.do?product=WOS&SID=D2ffI7nuzVvNS9FiYSC&search_mode=AdvancedSearch&replaceSetId=3&editState=init) |  |  |
|  | | | | | |
| # 2 | [**8,089**](http://apps.webofknowledge.com.ep.fjernadgang.kb.dk/summary.do?product=WOS&doc=1&qid=2&SID=D2ffI7nuzVvNS9FiYSC&search_mode=AdvancedSearch&update_back2search_link_param=yes) | TS=(magnetoencephalography)  *Indexes=SCI-EXPANDED, SSCI, A&HCI, CPCI-S, CPCI-SSH, BKCI-S, BKCI-SSH, ESCI, CCR-EXPANDED, IC Timespan=All years* | [Edit](http://apps.webofknowledge.com.ep.fjernadgang.kb.dk/WOS_AdvancedSearch_input.do?product=WOS&SID=D2ffI7nuzVvNS9FiYSC&search_mode=AdvancedSearch&replaceSetId=2&editState=init) |  |  |
|  | | | | | |
| # 1 | [**22,166**](http://apps.webofknowledge.com.ep.fjernadgang.kb.dk/summary.do?product=WOS&doc=1&qid=1&SID=D2ffI7nuzVvNS9FiYSC&search_mode=AdvancedSearch&update_back2search_link_param=yes) | TS=(electroencephalography)  *Indexes=SCI-EXPANDED, SSCI, A&HCI, CPCI-S, CPCI-SSH, BKCI-S, BKCI-SSH, ESCI, CCR-EXPANDED, IC Timespan=All years* |  |  |  |
